# Genome-wide association meta-analysis of human longevity identifies a novel locus conferring survival beyond 90 years of age

**DOI:** 10.1093/hmg/ddu139

**Published:** 2014-03-31

**Authors:** Joris Deelen, Marian Beekman, Hae-Won Uh, Linda Broer, Kristin L. Ayers, Qihua Tan, Yoichiro Kamatani, Anna M. Bennet, Riin Tamm, Stella Trompet, Daníel F. Guðbjartsson, Friederike Flachsbart, Giuseppina Rose, Alexander Viktorin, Krista Fischer, Marianne Nygaard, Heather J. Cordell, Paolina Crocco, Erik B. van den Akker, Stefan Böhringer, Quinta Helmer, Christopher P. Nelson, Gary I. Saunders, Maris Alver, Karen Andersen-Ranberg, Marie E. Breen, Ruud van der Breggen, Amke Caliebe, Miriam Capri, Elisa Cevenini, Joanna C. Collerton, Serena Dato, Karen Davies, Ian Ford, Jutta Gampe, Paolo Garagnani, Eco J.C. de Geus, Jennifer Harrow, Diana van Heemst, Bastiaan T. Heijmans, Femke-Anouska Heinsen, Jouke-Jan Hottenga, Albert Hofman, Bernard Jeune, Palmi V. Jonsson, Mark Lathrop, Doris Lechner, Carmen Martin-Ruiz, Susan E. Mcnerlan, Evelin Mihailov, Alberto Montesanto, Simon P. Mooijaart, Anne Murphy, Ellen A. Nohr, Lavinia Paternoster, Iris Postmus, Fernando Rivadeneira, Owen A. Ross, Stefano Salvioli, Naveed Sattar, Stefan Schreiber, Hreinn Stefánsson, David J. Stott, Henning Tiemeier, André G. Uitterlinden, Rudi G.J. Westendorp, Gonneke Willemsen, Nilesh J. Samani, Pilar Galan, Thorkild I.A. Sørensen, Dorret I. Boomsma, J. Wouter Jukema, Irene Maeve Rea, Giuseppe Passarino, Anton J.M. de Craen, Kaare Christensen, Almut Nebel, Kári Stefánsson, Andres Metspalu, Patrik Magnusson, Hélène Blanché, Lene Christiansen, Thomas B.L. Kirkwood, Cornelia M. van Duijn, Claudio Franceschi, Jeanine J. Houwing-Duistermaat, P. Eline Slagboom

**Affiliations:** 1 Department of Molecular Epidemiology,; 2 Netherlands Consortium for Healthy Ageing,; 3 Department of Medical Statistics and Bioinformatics,; 4 Department of Cardiology and; 5 Department of Gerontology and Geriatrics, Leiden University Medical Center, Leiden 2300 RC, The Netherlands; 6 Department of Epidemiology and; 7 Department of Internal Medicine, Erasmus Medical Center, Rotterdam 3000 CA, The Netherlands; 8 Institute of Genetic Medicine, International Centre for Life, Newcastle University, Newcastle upon Tyne NE1 3BZ, UK; 9 Epidemiology, Institute of Public Health and; 10 Department of Gynecology and Obstetrics, Institute of Clinical Research, University of Southern Denmark, Odense C DK-5000, Denmark; 11 Department of Clinical Genetics and; 12 Clinical Biochemistry and Pharmacology, Odense University Hospital, Odense C DK-5000, Denmark; 13 Fondation Jean Dausset-CEPH, Paris 75010, France; 14 Department of Medical Epidemiology and Biostatistics, Karolinska Institute, Stockholm SE-171 77, Sweden,; 15 Estonian Genome Center and; 16 Institute of Molecular and Cell Biology, University of Tartu, Tartu 51010, Estonia; 17 Population Genomics, deCODE Genetics, Reykjavík 101, Iceland; 18 Institute of Clinical Molecular Biology and; 19 Institute of Medical Informatics and Statistics, Christian-Albrechts-University, Kiel 24105, Germany; 20 Department of Biology, Ecology and Earth Science, University of Calabria, Rende 87036, Italy; 21 Delft Bioinformatics Lab, Delft University of Technology, Delft 2600 GA, The Netherlands; 22 Department of Cardiovascular Sciences, University of Leicester, Leicester LE3 9QP, UK; 23 National Institute for Health Research Leicester Cardiovascular Biomedical Research Unit, Glenfield Hospital, Leicester LE3 9QP, UK; 24 Human and Vertebrate Analysis and Annotation, The Wellcome Trust Sanger Institute, The Wellcome Trust Genome Campus, Hinxton, Cambridge CB10 1SA, UK; 25 School of Medicine, Dentistry and Biomedical Science, Queens University Belfast, Belfast BT9 7BL, UK; 26 Department of Psychiatry, University of Iowa, Iowa City, IA 52242, USA; 27 Department of Experimental, Diagnostic and Specialty Medicine and; 28 Interdepartmental Centre ‘L. Galvani’, University of Bologna, Bologna 40126, Italy; 29 Institute for Ageing and Health, Newcastle University, Campus for Ageing and Vitality, Newcastle upon Tyne NE4 5PL, UK; 30 Robertson Center for Biostatistics and; 31 Institute of Cardiovascular and Medical Sciences, University of Glasgow, Glasgow G12 8QQ, UK; 32 Laboratory of Statistical Demography, Max Planck Institute for Demographic Research, Rostock 18057, Germany; 33 Department of Biological Psychology, VU University Amsterdam, Amsterdam 1081 BT, The Netherlands; 34 EMGO Institute for Health and Care Research, VU University Medical Center, Amsterdam 1081 BT, The Netherlands; 35 Geriatrics, Landspitali University Hospital, Reykjavik 101, Iceland; 36 Faculty of Medicine, University of Iceland, Reykjavik 101, Iceland; 37 Institut de Génomique, CEA, Évry 91057, France; 38 McGill University and Génome Québec Innovation Centre, Montréal, Québec, CanadaH3A 1A4; 39 Cytogenetics Laboratory, Belfast Health and Social Care Trust, Belfast BT8 8BH, UK; 40 Estonian Biocentre, Tartu 51010, Estonia; 41 Section for Epidemiology, Department of Public Health, Aarhus University, Aarhus C DK-8000, Denmark; 42 MRC Centre for Causal Analyses in Translational Epidemiology, School of Social and Community Medicine, University of Bristol, Bristol BS8 2BN, UK; 43 Department of Neuroscience, Mayo Clinic, Jacksonville, FL 32224, USA; 44 BHF Glasgow Cardiovascular Research Centre, Faculty of Medicine, University of Glasgow, Glasgow G12 8TA, UK; 45 PopGen Biobank, Christian-Albrechts-University and University Hospital Schleswig-Holstein, Kiel 24105, Germany; 46 Department of Child and Adolescent Psychiatry, Erasmus Medical Center-Sophia Children's Hospital, Rotterdam 3000 CA, The Netherlands; 47 Université Sorbonne Paris Cité-UREN (Unité de Recherche en Epidémiologie Nutritionnelle), U557 Inserm; U1125 Inra; Cnam; Université Paris 13, CRNH IdF, Bobigny 93017, France; 48 Novo Nordisk Foundation Center for Basic Metabolic Research, Section on Metabolic Genetics, Faculty of Health and Medical Sciences, University of Copenhagen, Copenhagen N DK-2200, Denmark; 49 Institute of Preventive Medicine, Bispebjerg and Frederiksberg University Hospitals, Frederiksberg DK-2000, Denmark; 50 Interuniversity Cardiology Institute of the Netherlands, Utrecht 3501 DG, The Netherlands; 51 IRCCS Institute of Neurological Science, Bellaria Hospital, Bologna 40139, Italy; 52 CNR-ISOF, Bologna 40129, Italy

## Abstract

The genetic contribution to the variation in human lifespan is ∼25%. Despite the large number of identified disease-susceptibility loci, it is not known which loci influence population mortality. We performed a genome-wide association meta-analysis of 7729 long-lived individuals of European descent (≥85 years) and 16 121 younger controls (<65 years) followed by replication in an additional set of 13 060 long-lived individuals and 61 156 controls. In addition, we performed a subset analysis in cases aged ≥90 years. We observed genome-wide significant association with longevity, as reflected by survival to ages beyond 90 years, at a novel locus, rs2149954, on chromosome 5q33.3 (OR = 1.10, *P* = 1.74 × 10^−8^). We also confirmed association of rs4420638 on chromosome 19q13.32 (OR = 0.72, *P* = 3.40 × 10^−36^), representing the *TOMM40*/*APOE*/*APOC1* locus. In a prospective meta-analysis (*n* = 34 103), the minor allele of rs2149954 (T) on chromosome 5q33.3 associates with increased survival (HR = 0.95, *P* = 0.003). This allele has previously been reported to associate with low blood pressure in middle age. Interestingly, the minor allele (T) associates with decreased cardiovascular mortality risk, independent of blood pressure. We report on the first GWAS-identified longevity locus on chromosome 5q33.3 influencing survival in the general European population. The minor allele of this locus associates with low blood pressure in middle age, although the contribution of this allele to survival may be less dependent on blood pressure. Hence, the pleiotropic mechanisms by which this intragenic variation contributes to lifespan regulation have to be elucidated.

## INTRODUCTION

Worldwide, human life expectancy has increased remarkably over the last two centuries (1), although the healthy life expectancy lags behind. Citizens of the European Union, for example, spend only 75–80% of their lifespan in good health (2). Families in which longevity clusters form an exception in this sense, by showing beneficial or ‘youthful’ profiles for many metabolic and immune-related parameters (3–7) and a low prevalence of common diseases from middle age onwards (5,8,9). Therefore, the genome of long-lived individuals is investigated to identify variants that promote healthy aging and protect against age-related disease. This is a major challenge because the genetic component of lifespan variation in the population at large has been estimated to be only ∼25% (10,11) and is assumed to be determined by many, still uncharacterized, genes (12,13). Genetic influences on human longevity are expected to reflect longevity assurance mechanisms acting across species (14), as well as more heterogeneous population-specific effects. Although numerous genome-wide association studies (GWAS) have successfully identified loci involved in common, age-related diseases (15), the corresponding susceptibility loci do not explain the genetic component of human longevity (16). GWAS for human longevity have thus far failed to identify genome-wide significant loci, besides the well-known *TOMM40/APOE/APOC1* locus (17–19).

In this paper, we conducted a large genome-wide association meta-analysis of human longevity in 14 studies with long-lived cases (≥85 years) and younger controls (<65 years) from European descent. In addition, we performed a subset analysis in cases aged ≥90 years. The novel longevity locus we identified was tested for association with prospective (cause-specific) mortality in a meta-analysis of 11 European cohorts and examined for association with various metabolic traits that may explain the mechanism by which the locus contributes to survival to high ages.

## RESULTS

### Genome-wide association analysis

**Figure 1.**
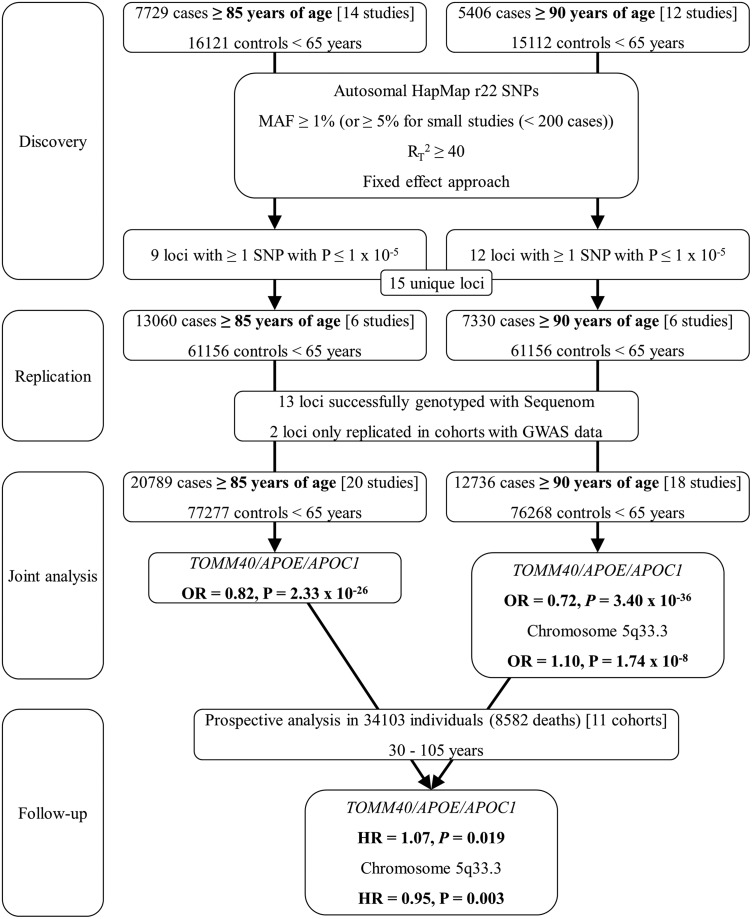
Flow chart of experimental work. The analysis in the cases aged ≥90 years is a subset analysis of the analysis in the cases aged ≥85 years. Twelve out of 14 studies used for the discovery phase analysis of cases aged ≥85 years contained at least 100 cases over 90 years of age and were thus analyzed in the subset analysis of cases aged ≥90 years.

In order to identify novel loci involved in lifespan regulation, we conducted a meta-analysis on GWAS data of 7729 long-lived cases (≥85 years) and 16 121 younger controls (<65 years) from 14 studies originating from 7 European countries (Supplementary Material, Table S1). For each study, cases and controls originated from the same country. Given the higher heritability of longevity at older ages (11,20), we performed a subset analysis in which we compared cases aged ≥90 years (*n* = 5406) with 15 112 controls (<65 years) from the corresponding control cohorts. Replication was performed in 13 060 cases aged ≥85 years (of which 7330 were ≥90 years) and 61 156 controls from 6 additional studies, of which 3 originated from European countries not represented in the discovery phase meta-analysis (Supplementary Material, Table S1). Analysis of each study was performed using a logistic regression-based method, and results were adjusted for study-specific genomic inflation factors (*λ*) (Supplementary Material, Table S2). Meta-analysis was performed on 2 480 356 (≥85 years) and 2 470 825 (≥90 years) imputed SNPs using a fixed-effect approach, and results were further adjusted for the overall genomic inflation factor (*λ* = 1.019) (Supplementary Material, Fig. S1). A flow chart of the consecutive analysis steps is depicted in Figure 1.

**Figure 2.**
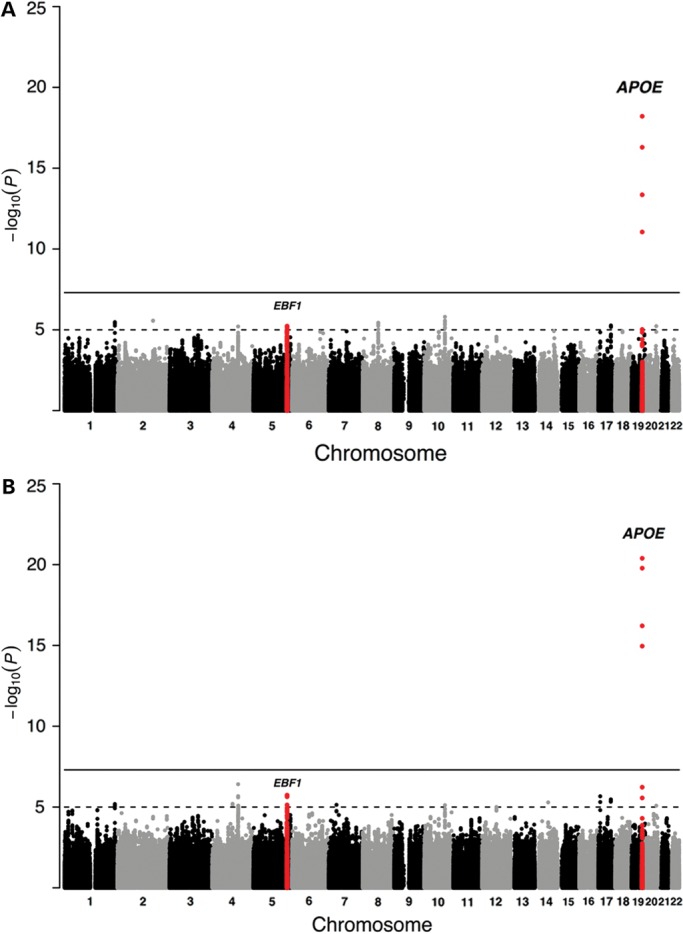
Results of the discovery phase analysis. Manhattan plot presenting the −log_10_ *P*-values from the discovery phase analysis of cases aged ≥85 years (**A**) and ≥90 years (**B**). The loci that showed a genome-wide significant association after the joint analysis of the discovery and replication phase (chromosome 19q13.32 and 5q33.3) are shown in red.

**Figure 3.**
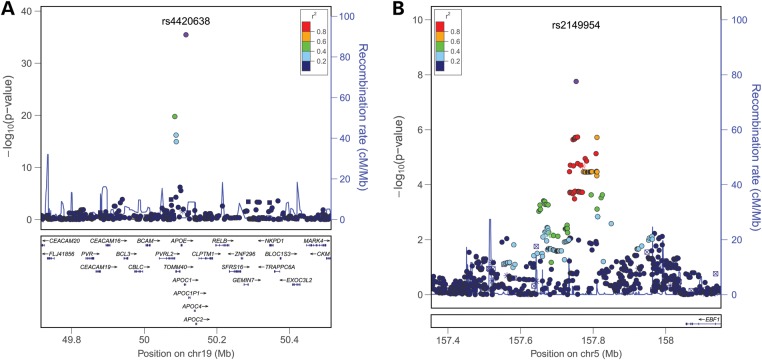
Regional association plots for the chromosome 19q13.32 and 5q33.3 loci. Results of the discovery-phase analysis of chromosome 19q13.32 (**A**) and 5q33.3 (**B**) in cases aged ≥90 years, generated using LocusZoom (http://csg.sph.umich.edu/locuszoom/) (22). For the two SNPs taken forward to the replication phase (rs4420638 and rs2149954), the results of the joint analysis are plotted. The color of the SNPs is based on the LD with the lead SNP (shown in purple). The blue peaks represent the recombination rates based on HapMap Phase I+II CEU release 22 (hg18/build36), and the RefSeq genes in the region are shown in the lower panel.

**Figure 4.**
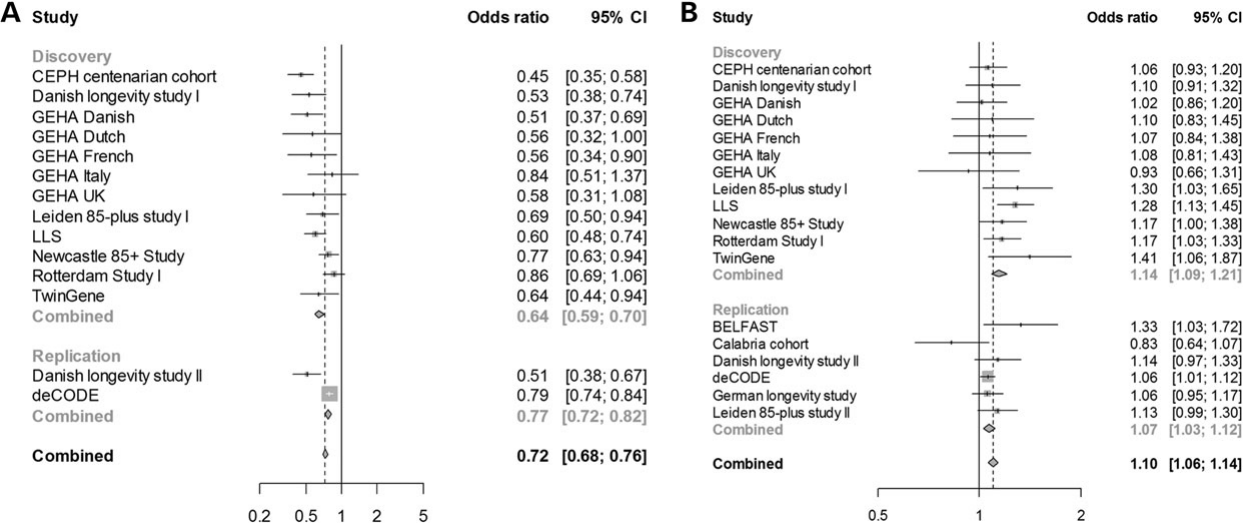
Forest plots for rs4420638 and rs2149954. Forest plots representing the odds ratios with 95% CI of rs4420638 (**A**) and rs2149954 (**B**) for the cohorts analyzed in the discovery and replication phase (≥90 years). The size of the boxes represents the sample size of the cohort.

The discovery phase meta-analyses of the cases aged ≥85 years (*n* = 7729) showed genome-wide significant association with survival into old age at one locus, the previously identified *TOMM40/APOE/APOC1* locus (17,21) (rs4420638 (G); odds ratio (OR) = 0.71, *P* = 6.14 × 10^−19^; Table 1). No gender-dependent effects were observed in the sex-stratified analysis of the cases aged ≥85 years (Supplementary Material, Table S4). The discovery-phase meta-analysis of the cases aged ≥90 years (*n* = 5406) showed a similar result, i.e. the *TOMM40/APOE/APOC1* locus was the only genome-wide significant locus (OR = 0.64, *P* = 4.09 × 10^−21^; Fig. 2 and Table 2). The regional association plot and forest plot for the *TOMM40/APOE/APOC1* locus are depicted in Figures 3 and 4, respectively. Although several SNPs on chromosome 19q13.32, which are in moderate linkage disequilibrium (LD) with rs4420638, show additional association with survival into old age, meta-analysis conditional on rs4420638 showed no independent associations among these SNPs (Supplementary Material, Fig S2 and Table S3).

**Table 1.** Results of the discovery phase, replication phase and joint analysis of cases aged ≥85 years

Locus	Lead SNP	Chromosome	Position	Candidate/closest gene	EA	Analysis	*n*		EAF		OR	95% CI	*P*	*I*^2^ (%)	*P*_het_
							Cases	Controls	Cases	Controls					
1q43	**rs1625040**	1	235 213 002	*MTR, RYR2*	A	Discovery	7729	16 121	0.170	0.150	1.16	1.09–1.23	3.36 × 10^−6^		
						Replication	13 027	60 914	0.178	0.182	1.02	0.98–1.07	0.216		
						Joint	20 756	77 035			1.07	1.03–1.10	3.50 × 10^−4^	31.0	0.093
2q24.3	rs6432832	2	166 079 072	*CSRNP3*	A	Discovery	7729	16 121	0.344	0.321	1.12	1.07–1.17	2.79 × 10^−6^		
						Replication	13 019	60 824	0.346	0.339	1.03	1.00–1.07	0.029		
						Joint	20 748	76 945			1.06	1.03–1.09	8.73 × 10^−6^	0.0	0.467
4q27	**rs13114426**	4	120 942 533	*PDE5A, MAD2L1*	T	Discovery	7729	16 121	0.387	0.405	0.90	0.87–0.95	2.20 × 10^−5^		
						Replication	13 024	60 932	0.364	0.351	1.00	0.97–1.04	0.711		
						Joint	20 753	77 053			0.97	0.94–0.99	0.033	46.5	0.012
5q33.3	**rs2149954**	5	157 753 180	*EBF1*	T	Discovery	7729	16 121	0.388	0.360	1.12	1.07–1.17	5.98 × 10^−6^		
						Replication	12 973	60 262	0.365	0.352	1.04	1.01–1.07	0.013		
						Joint	20 702	76 383			1.07	1.04–1.09	4.34 × 10^−6^	28.2	0.118
8q13.3	rs10957550^a^	8	72 457 142	*EYA1*	A	Discovery	7727	16 093	0.268	0.285	0.88	0.84–0.93	3.61 × 10^−6^		
						Replication	10 056	56 262	0.236	0.244	0.95	0.92–0.99	0.012		
						Joint	17 783	72 355			0.92	0.90–0.95	1.41 × 10^−6^	29.4	0.130
10q23.33	**rs4466755**	10	96 622 243	*CYP2C19, CYP2C9*	T	Discovery	7729	16 121	0.454	0.443	1.12	1.07–1.16	2.72 × 10^−6^		
						Replication	13 051	61 105	0.488	0.508	0.98	0.95–1.01	0.129		
						Joint	20 780	77 226			1.03	1.00–1.05	0.161	65.6	2.15 × 10^−5^
17q23.3	rs17760362	17	58 772 399	*TANC2*	A	Discovery	7729	16 121	0.252	0.233	1.13	1.07–1.19	5.38 × 10^−6^		
						Replication	13 007	60 679	0.252	0.249	1.04	1.00–1.07	0.033		
						Joint	20 736	76 800			1.07	1.04–1.10	1.56 × 10^−5^	0.0	0.473
19q13.32	**rs4420638**^a^	19	50 114 786	*APOE*	G	Discovery	7728	16 111	0.157	0.195	0.71	0.67–0.77	6.14 × 10^−19^		
						Replication	10 165	57 126	0.180	0.202	0.87	0.83–0.91	2.12 × 10^−12^		
						Joint	17 893	73 237			0.82	0.79–0.85	2.33 × 10^−26^	80.2	4.35 × 10^−10^
20q13.2	**rs8126377**	20	51 590 254	*TSHZ2, ZNF217*	G	Discovery	7532	15 902	0.059	0.069	0.79	0.71–0.87	1.35 × 10^−5^		
						Replication	12 974	60 647	0.058	0.054	1.01	0.94–1.08	0.901		
						Joint	20 506	76 549			0.93	0.88–0.99	0.020	51.1	0.006

EA, effect allele; EAF, effect allele frequency after pooling the data of all analyzed individuals; OR, odds ratio for the effect allele; 95% CI, 95% confidence interval; *I*^2^, heterogeneity statistic; *P*_het_, *P*-value for heterogeneity.

^a^Genotyping of these SNPs with the Sequenom MassARRAY system for the replication phase was unsuccessful. The SNPs in bold overlap with Table 2.

**Table 2.** Results of the discovery phase, replication phase and joint analysis of cases aged ≥90 years

Locus	Lead SNP	Chromosome	Position	Candidate/closest gene	EA	Analysis	*n*		EAF		OR	95% CI	*P*	*I*^2^ (%)	*P*_het_
							Cases	Controls	Cases	Controls					
1q43	**rs1625040**	1	235 213 002	*MTR, RYR2*	A	Discovery	5406	15 112	0.176	0.150	1.18	1.10–1.26	6.53 × 10^−6^		
						Replication	7310	60 914	0.175	0.182	1.05	0.99–1.10	0.065		
						Joint	12 716	76 026			1.10	1.05–1.14	2.60 × 10^−5^	9.3	0.343
4q22.2	rs4693331	4	94 760 609	*GRID2*	C	Discovery	5406	15 112	0.416	0.444	0.89	0.84–0.93	6.63 × 10^−6^		
						Replication	7267	60 324	0.449	0.440	1.03	0.99–1.07	0.095		
						Joint	12 673	75 436			0.97	0.94–1.00	0.139	61.3	3.51 × 10^−4^
4q27	**rs13114426**	4	120 942 533	*PDE5A, MAD2L1*	T	Discovery	5406	15 112	0.381	0.405	0.88	0.84–0.92	2.11 × 10^−6^		
						Replication	7305	60 932	0.369	0.351	0.98	0.94–1.02	0.336		
						Joint	12 711	76 044			0.94	0.91–0.97	1.95 × 10^−4^	32.5	0.090
5q33.3	**rs2149954**	5	157 753 180	*EBF1*	T	Discovery	5406	15 112	0.396	0.360	1.14	1.09–1.21	1.85 × 10^−6^		
						Replication	7298	60 262	0.374	0.352	1.07	1.03–1.12	5.98 × 10^−4^		
						Joint	12 704	75 374			1.10	1.06–1.14	1.74 × 10^−8^	28.5	0.125
7p14.2	rs11977641	7	36 761 949	*AOAH, ELMO1*	C	Discovery	5406	15 112	0.062	0.076	0.78	0.70–0.87	7.31 × 10^−6^		
						Replication	3049	4805	0.071	0.073	0.93	0.82–1.06	0.226		
						Joint	8455	19 917			0.84	0.77–0.91	1.57 × 10^−5^	50.2	0.010
10q23.33	**rs4466755**	10	96 622 243	*CYP2C19, CYP2C9*	T	Discovery	5406	15 112	0.455	0.445	1.13	1.07–1.18	1.30 × 10^−5^		
						Replication	7326	61 105	0.477	0.508	0.98	0.94–1.02	0.208		
						Joint	12 732	76 217			1.03	1.00–1.07	0.087	55.4	0.002
12q15	rs11834614	12	67 197 344	*MDM1, RAP1B*	C	Discovery	5406	15 112	0.138	0.155	0.85	0.79–0.91	9.94 × 10^−6^		
						Replication	7272	60 210	0.165	0.173	1.01	0.96–1.07	0.603		
						Joint	12 678	75 322			0.95	0.91–0.99	0.023	43.9	0.024
14q23.2	rs2784505	14	61 501 766	*SYT16*	G	Discovery	5406	15 112	0.080	0.067	1.23	1.11–1.35	8.87 × 10^−5^		
						Replication	7323	60 979	0.070	0.066	1.10	1.02–1.19	0.012		
						Joint	12 729	76 091			1.15	1.08–1.22	9.47 × 10^−6^	28.3	0.127
17p13.1	rs940850	17	8 870 805	*NTN1*	T	Discovery	5405	15 112	0.072	0.093	0.78	0.70–0.87	4.93 × 10^−6^		
						Replication	7276	60 146	0.109	0.118	1.03	0.97–1.10	0.318		
						Joint	12 681	75 258			0.95	0.90–1.01	0.111	63.7	1.32 × 10^−4^
17q23.2	rs2109265	17	58 307 001	*MARCH10, TANC2*	A	Discovery	5406	15 112	0.443	0.420	1.13	1.08–1.19	3.34 × 10^−6^		
						Replication	7307	60 672	0.453	0.465	1.01	0.97–1.05	0.671		
						Joint	12 713	75 784			1.06	1.02–1.09	0.001	34.7	0.074
19q13.32	**rs4420638**^a^	19	50 114 786	*APOE*	G	Discovery	5405	15 102	0.145	0.195	0.64	0.59–0.70	4.09 × 10^−21^		
						Replication	4861	57 126	0.165	0.202	0.77	0.72–0.82	2.95 × 10^−18^		
						Joint	10 266	72 228			0.72	0.68–0.76	3.40 × 10^−36^	70.1	3.69 × 10^−5^
20q13.2	**rs8126377**	20	51 590 254	*TSHZ2, ZNF217*	G	Discovery	5209	14 893	0.057	0.068	0.75	0.66–0.85	3.38 × 10^−5^		
						Replication	7278	60 647	0.063	0.054	1.04	0.95–1.13	0.309		
						Joint	12 487	75 540			0.94	0.87–1.00	0.117	58.1	0.001

EA, effect allele; EAF, effect allele frequency after pooling the data of all analyzed individuals; OR, odds ratio for the effect allele; 95% CI, 95% confidence interval; *I*^2^, heterogeneity statistic; *P*_het_, *P*-value for heterogeneity.

^a^Genotyping of this SNP with the Sequenom MassARRAY system for the replication phase was unsuccessful. The SNPs in bold overlap with Table 1.

### Replication

In addition to the *TOMM40/APOE/APOC1* locus, we found eight loci that showed suggestive evidence for association in the discovery-phase meta-analysis of cases aged ≥85 years (*P* ≤ 1 × 10^−5^; Table 1), whereas six additional SNPs met this criterion in the meta-analysis of cases aged ≥90 years (Table 2). The most or (when not successfully measured) second most significant SNPs from these 14 loci and the *TOMM40/APOE/APOC1* locus were taken forward for replication in 13 060 cases aged ≥85 years (of which 7330 were also ≥90 years) and 61 156 controls from 6 additional studies. In the joint analysis of the discovery and replication phase of the cases aged ≥85 years (9 loci), the *TOMM40/APOE/APOC1* locus remained the only genome-wide significant locus (Table 1). The joint analysis of the discovery and replication phase of the cases aged ≥90 years (12 loci), however, showed an additional genome-wide significant locus, rs2149954 (T), on chromosome 5q33.3 (OR = 1.10, *P* = 1.74 × 10^−8^; Table 2). Although the association of this SNP with survival up to 85 years is not genome-wide significant (OR = 1.07, *P* = 4.34 × 10^−6^; Table 1), the locus likely affects survival from middle age onwards. The regional association plot (based on the discovery phase only) and forest plot of this locus are depicted in Figures 3 and 4, respectively. Conditional analysis of rs4420638 in the discovery phase studies showed that the association of rs2149954 (T) with survival is independent of the *TOMM40*/*APOE*/*APOC1* locus (*P* = 7.20 × 10^−6^ instead of *P* = 5.98 × 10^−6^ in the analysis of survival up to 85 years).

### Prospective analysis

To determine the association of rs4420638 (*TOMM40/APOE/APOC1* locus) and rs2149954 (chromosome 5q33.3 locus) with longitudinal survival, we performed a prospective meta-analysis of the 2 SNPs in 34 103 individuals aged 30–105 years from 11 different cohorts, of which 8582 had died after a mean follow-up time ranging from 2.2 to 17.4 years (Supplementary Material, Table S5). Carriers of the minor allele of rs4420638 (G) showed significantly higher all-cause mortality (hazard ratio (HR) = 1.07, *P* = 0.019), whereas carriers of the minor allele of rs2149954 (T) demonstrated significantly lower all-cause mortality (HR = 0.95, *P* = 0.003; Supplementary Material, Table S6).

### Association with cardiovascular disease and blood pressure

To gain insight into the mechanism by which the chromosome 5q33.3 locus might promote human longevity, we analyzed the cause-specific mortality of rs2149954. Carriers of the minor allele of rs2149954 have a lower mortality risk for cardiovascular disease (CVD) (HR = 0.86, *P* = 0.004), which mainly appeared to be caused by protection from stroke (HR = 0.60, *P* = 2.27 × 10^−7^). In addition, we observed an effect of this SNP on non-CVD mortality (HR = 0.86, *P* = 0.002) (Supplementary Material, Table S7). We also examined the Coronary ARtery DIsease Genome-Wide Replication And Meta-Analysis (CARDIoGRAM) GWAS (23), which showed a significant association of rs2149954 with a decreased risk for coronary artery disease (CAD) (OR = 0.96, *P* = 0.011) (Supplementary Material, Table S8). In addition, two SNPs on chromosome 5q33.3 in high LD with rs2149954, rs9313772 (*r*^2^ = 0.928) and rs11953630 (*r*^2^ = 0.854) have previously been reported to associate with blood pressure and hypertension (24,25). As expected, examining rs2149954 in the International Consortium for Blood Pressure GWAS (24) showed a significant association of the minor allele with lower diastolic (*P* = 3.46 × 10^−5^) and systolic (*P* = 6.55 × 10^−6^) blood pressure (Supplementary Material, Table S9). Despite the highly interesting association of the minor allele of rs2149954 with low blood pressure and a decreased risk for CAD, stroke and mortality, its association with decreased all-cause mortality was not influenced by blood pressure in two studies of participants aged ≥75 years (PROSPER and Leiden 85-plus study Cohort II; Supplementary Material, Table S10). This may indicate that at higher ages, this locus influences longevity via pathways additional to those involved in blood pressure regulation.

### Phenotypic characterization and pathway analysis

In an attempt to identify the underlying mechanism by which this novel longevity locus at chromosome 5q33.3 could influence human longevity, we examined rs2149954 in the published data of several large GWAS consortia for association with metabolic traits in generally middle-aged individuals. None of the investigated traits, i.e. 2 h glucose (OGTT), Hb_1_Ac, fasting glucose, fasting insulin, insulin resistance (HOMA-IR), β-cell activity (HOMA-B), total/HDL/LDL cholesterol, triglycerides and type 2 diabetes (26–32), demonstrated evidence for association (all *P* > 0.05) with rs2149954 (Supplementary Material, Tables S8 and S9).

Gene set enrichment analysis (GSEA) of the meta-analysis results of the discovery-phase analysis of survival aged ≥90 years using Meta-Analysis Gene-set Enrichment of variaNT Associations (MAGENTA) (33), as well as examination of interconnectivity of implicated genes using Gene Relationships Across Implicated Loci (GRAIL) (34) (Supplementary Material, Fig. S3 and Table S11), provided no firm clues for potential pathways involved in human longevity.

### Fine mapping and functional characterization

**Figure 5.**
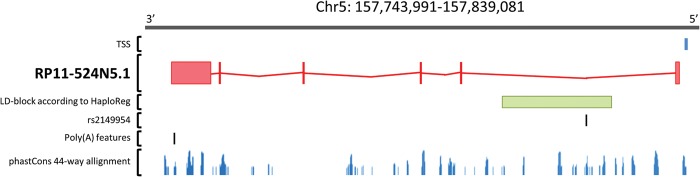
Chromosomal region around rs2149954. The region contains a lincRNA (RP11-524N5.1) for which the poly(A) features are supported by PolyA-seq reads from liver, muscle and testis. RP11-524N5.1 is transcribed from the negative strand, and the phastCons 44-way alignment supports conservation of the TSS, 3′ UTR and the third, fifth and last exon of the transcript. Rs2149954 and the 25 SNPs in high LD (*r*^2^ ≥ 0.8, according to HaploReg v2 (35)) are located in the first intron of RP11-524N5.1.

The newly identified longevity locus on chromosome 5q33.3 is located in an intergenic region on chromosome 5q33.3, 302 kb downstream of the *EBF1* gene. To determine the functional impact of this locus, we first identified the SNPs in LD with rs2149954 (*r*^2^ ≥ 0.8) using the 1000 Genomes CEU Phase 1 data implemented in HaploReg v2 (http://www.broadinstitute.org/mammals/haploreg/haploreg.php) (35). In total, we identified 25 SNPs, spanning a region of ∼22.3 kb (Supplementary Material, Table S12). Subsequently, we examined the potential effects of these SNPs on gene expression using several eQTL databases. None of the SNPs showed an association with gene expression in the various examined tissues, so it is still unclear in which tissue(s) the locus exert its longevity-promoting effect. We did, however, find some promising functional implication of this locus, i.e. the presence of multiple DNase I hypersensitivity sites, transcription factor binding sites and enhancer histone marks, by exploring ENCODE data using HaploReg v2 (35) and RegulomeDB (http://www.regulomedb.org/) (36) (Supplementary Material, Table S12). Very recently, a large intergenic non-coding RNA (lincRNA), RP11-524N5.1, has been annotated right on top of our locus. The poly(A) features of this lincRNA are supported by PolyA-seq reads from liver, muscle and testis. PhastCons 44-way alignment supports conservation of the transcription start site (TSS), 3′ UTR and the third, fifth and last exon of the lincRNA transcript (Fig. 5). The transcript does not align to the mouse genome, but orthologous transcripts are found in other primate genome sequences, suggesting that this is a primate-specific lincRNA.

## DISCUSSION

We have performed the largest genome-wide association meta-analysis for human longevity, in which a novel locus on chromosome 5q33.3 associating with survival beyond 90 years was identified.

The minor allele of rs2149954 (T) promotes human longevity by reducing the risk of mortality owing to stroke and non-cardiovascular causes. In addition, this allele has previously been associated with low blood pressure, which may explain the protection from CVD mortality risk in middle age. At ages above 80 years, however, low SBP associates with increased mortality (37,38). Hence, the observed blood pressure-independent association of the minor allele with mortality ≥75 years may be due to pleiotropic effects on other mortality-related clinical parameters. Examination of publically available data of several large GWAS consortia for association of the locus with parameters related to glucose and fat metabolism provided as yet no clues for other potentially involved mechanisms.

Rs2149954 is located in an intergenic region on chromosome 5q33.3 between *CLINT1* and *EBF1*. The presence of several regulatory elements in this region implies that transcription factor binding and/or expression of (nearby) genes could be influenced. The currently available eQTL databases did not provide evidence for such effects, which might be due to the limited tissue diversity of the databases. The effects of the chromosome 5q33.3 locus on human longevity might be exerted through the lincRNA, which has recently been annotated right on top of our locus (RP11-524N5.1) and shows evidence for expression in liver, muscle and testis. LincRNAs are involved in chromatin modification and transcriptional regulation (39) and seem to play a role in human disease (40). However, the newly annotated lincRNA is not yet available in the large eQTL databases, and the effect of SNPs in the chromosome 5q33.3 locus on expression of this transcript still needs to be determined. Hence, further functional studies are required to illuminate the mechanism by which this locus influences human longevity.

GWAS has thus far not been a successful approach to identify genome-wide significant hits for human longevity or mortality besides the well-known *TOMM40/APOE/APOC1* locus (17–19). The *FOXO3A* locus, for which the longevity effect is most prominent in individuals aged ≥100 years (41), showed only moderate evidence for association with survival ≥90 years in the discovery phase of our GWAS (lowest *P* = 1.35 × 10^−4^ (rs1268161)). Sebastiani and colleagues suggested that human longevity might be explained by a signature consisting of 281 SNPs (42). However, none of the SNPs (except the already known SNP rs2075650 in *TOMM40*) was significant after adjustment for multiple testing (*P* < 1.78 × 10^−4^ (0.05/281)). In addition, we did not observe an enrichment of significant SNPs from their signature in our data (*λ* = 1.004, Supplementary Material, Fig. S4). Because the association of SNPs other than the *TOMM40/APOE/APOC1* locus could not be replicated in this, much larger, GWAS, we have doubts that these signature SNPs are indeed candidate SNPs influencing human longevity. Although we detected merely one novel genome-wide significant locus, the current GWAS had sufficient power, based on our results, to detect lifespan-regulating loci with relatively small effects (OR <0.9 and >1.1).

The genetic component of human longevity is small (∼25%) (10,11) and is assumed to be determined by many genes (12,13). Furthermore, the genetic heterogeneity in ageing and lifespan regulation is expected to be high, because individual genes may contribute by a diversity of late acting deleterious stochastic (germline) variation resulting in a genetic component that is hard to disentangle (13). GWAS of complex late-onset diseases, such as osteoarthritis and Alzheimer's disease, with sample sizes comparable to our current study (43–45), have identified more loci compared with GWAS of longevity. This most likely reflects the greater inherent complexity of the longevity trait, with its diverse spectrum of biological pathways subject to intrinsic and extrinsic (environmental) interactions. Hence, even larger GWAS (>50 000 long-lived individuals) may be required to identify additional longevity loci, preferably in the most stringent phenotype, i.e. the oldest old.

As survival to ages ≥85 or 90 years is relatively common in Western populations, the human longevity trait suffers from etiological heterogeneity. Lifespan extension in the past generations owing to non-genetic factors likely created phenocopies diluting the genetic component of survival to ages ≥85 years. The genetic contribution to survival to ages ≥100 years is higher but will render smaller sample sizes for GWAS. This may explain why the novel locus on chromosome 5q33.3 was only genome-wide significant in the subset analysis of cases aged ≥90 years. For the same reason, a large number of individuals from the control groups (up to 50%, depending on the gender and year of birth of the individuals and demography of the cohort) will live to ages ≥85 years. In 2011, the mean life expectancy at age 65 in Europe was 21.3 years for women and 17.8 years for men (http://epp.eurostat.ec.europa.eu/portal/page/portal/product_details/dataset?P_product_code=TSDDE210), which makes selection of proper controls a challenging issue. The most ideal controls would be individuals from the same birth cohort as the long-lived cases that survived to the mean age of death of that birth cohort. However, for most of these individuals there is no DNA available. Alternatively, we selected controls that have not yet reached the age of 65 years at inclusion to represent the frequency of variants in the general population and minimize selection owing to mortality. Hence, the low contrast between cases and controls likely has reduced our probability of identifying longevity loci.

In addition, there will be differences between case and control cohorts that may have had an impact on our results. An example of a potential confounder is smoking behavior, which was not adequately measured in most elderly cohorts. However, none of the SNPs that were previously associated with smoking behavior in cohorts from European descent (according to the NHGRI GWAS Catalog (http://www.genome.gov/gwastudies/)), namely rs1051730, rs1329650 and rs4105144, show differences between cases (≥85 years) and controls in the joint analysis of the discovery and replication phase (all *P* > 0.05). We have to note that these SNPs only explain a small proportion of the variance observed in smoking behavior. However, as the frequency of these proxy SNPs for smoking behavior is similar between cases and controls, we expect no obvious differences in smoking behavior between the groups.

In conclusion, besides the previously implicated *TOMM40/APOE/APOC1* locus, we identified a novel locus on chromosome 5q33.3 that associates with survival beyond 90 years. Although rs2149954 is associated with survival beyond 90 years at a genome-wide significant level in our study, replication in additional cohorts from European as well as non-European descent is warranted. The minor allele of the lead SNP at this locus, rs2149954, promotes human longevity in a prospective meta-analysis by lowering the risk of mortality owing to stroke and non-cardiovascular causes. The locus harbors a lincRNA and is implicated in blood pressure regulation, but the mechanism by which it influences longevity likely also involves other traits.

## MATERIALS AND METHODS

### Study populations

The discovery analysis was performed in 7729 cases that survived to ages ≥85 years (of which 5406 also survived to ages ≥90 years) and 16 121 controls below 65 years at baseline, from 14 studies. Replication was performed in 13 060 cases that survived to ages ≥85 years (of which 7330 also survived to ages ≥90 years) and 61 156 controls below 65 years at baseline, from 6 additional studies. All individuals were of European descent. The details of the discovery and replication studies can be found in Supplementary Material, Tables S1 and S2. Some cohorts only provided controls (GOYA, NTR, SU.VI.MAX, TwinsUK and WTCCC2) or only cases (BELFAST, CEPH centenarian cohort, Danish longevity study I/II, Leiden 85-plus Study I/II and Newcastle 85+ Study), whereas others contained both (Calabria cohort, deCODE, EGCUT, GEHA Study, German longevity study, Leiden Longevity Study, Rotterdam Study I/II and TwinGene). The names of the studies in the tables and figures are based on the names of the cohorts containing the cases. The cases and controls used for each study originated from the same country (Supplementary Material, Table S1). The only exception is BELFAST (Northern Ireland), for which we used controls from the NTR (Netherlands). A check in the PROSPER study, which includes individuals from Northern Ireland and the Netherlands, showed that the allele frequencies in control individuals from both countries are similar for our SNPs (data not shown). All participants provided written informed consent, and the study was approved by the relevant institutional review boards.

### Genotyping, imputation and genome-wide association analysis

All discovery studies were genotyped using Illumina genotyping arrays, and pre-imputation quality control was performed for each study separately. Imputation was performed using IMPUTE or MACH with reference HapMap Phase I+II CEU release 22 (hg18/build36). Further details about the genotyping, quality control and imputation of each study are summarized in Supplementary Material, Table S2.

Two replication studies (deCODE and the Danish longevity study II) were also genotyped using Illumina genotyping arrays and imputed using IMPUTE with reference HapMap Phase I+II CEU release 22 (hg18/build36) (Danish longevity study II) or deCODE software (deCODE). The other replication studies were genotyped with the Sequenom MassARRAY system using iPLEX Gold genotyping assays (Sequenom, San Diego, CA, USA). More information about the studies used in the replication phase can be found in Supplementary Material, Tables S1 and S2. Of the 15 SNPs measured with the Sequenom MassARRAY system, 13 were successfully genotyped in at least 95% of the samples and the average genotyping call rate was 99.80%. We also checked the concordance between the SNPs measured with the Sequenom MassARRAY system and (imputed) GWAS data of the Leiden 85-plus study I cases, and the average concordance rate was 99.07%. The two SNPs that were not successfully genotyped with the Sequenom MassARRAY system (rs10957550 and rs4420368) were only analyzed in the replication studies, which had imputed GWAS data available (deCODE and the Danish longevity study II).

All studies were analyzed separately using CC-assoc (https://www.msbi.nl/dnn/Research/Genetics/Software/TestsforGWASinrelatedindividuals(cc_assoc).aspx), which is based on a modified version of the score test that takes into account imputation uncertainty and familial relatedness (46). SNPs with a low imputation quality $\left(R_{T}^{2}\leq 40\right)$ and a MAF of ≤1 or ≤5% (if *n*_cases_ < 200) were excluded from analysis in the discovery phase. Adjustment for population stratification of the discovery studies was performed by multiplying the $R_{T}^{2}$-adjusted variances of the score statistic with the genomic inflation factor (*λ*_range_ = 0.97 – 1.08, Supplementary Material, Table S2) of the study.

### Meta-analyses

For the meta-analyses, a fixed-effect approach was used. Scores and variances of the studies were combined to obtain a single meta-statistic, which was adjusted using the genomic inflation factor (*λ* = 1.019, discovery phase only) (Supplementary Material, Fig. S1). For each analysis, we only used studies with at least 100 cases (Supplementary Material, Table S1). *P*-values <5 × 10^−8^ were considered genome-wide significant (47). To determine heterogeneity across the studies, the between-study variance was calculated.

### Conditional analysis

To ascertain independent signals at the chromosome 19q13.32 locus, we performed a meta-analysis conditional on rs4420638 in all studies used for the discovery phase analysis in cases aged ≥85 years. The results are depicted in Supplementary Material, Figure S2 and Table S3.

### Sex-stratified analysis

Sex-stratified analysis of the cases aged ≥85 years (*n*_women_ = 5400 and *n*_men_ = 1865) was performed to investigate the presence of gender-dependent associations. In addition, the 15 loci that showed (suggestive) evidence for association with survival ≥85 and/or ≥90 years were tested for differences between sexes using the formula: $(\beta_{\mathrm{women}}-\beta_{\mathrm{men}})/\sqrt{\left({\text{SE}}_{\mathrm{women}}^{2}+{\text{SE}}_{\mathrm{men}}^{2}\right)}.$ The results of this analysis are depicted in Supplementary Material, Table S4.

### Prospective analysis

Prospective analysis of rs2149954 and rs4420638 was performed using a Cox proportional hazards model adjusted for age at baseline, sex and study-specific covariates. The details about each of the analyzed cohorts are summarized in Supplementary Material, Table S5.

### Pathway analysis

For the pathway analysis, we used GSEA implemented in MAGENTA (http://www.broadinstitute.org/mpg/magenta/) (33). In short, each SNP is mapped to a gene considering a window of 110 kb upstream and 40 kb downstream around the genes. Subsequently, each gene is assigned a gene association score based on the SNP with the lowest *P*-value, which is mapped to that gene and this score is adjusted for confounding factors like gene size and the amount of SNPs per kb. Genes within the HLA region were removed from analysis owing to high LD and high gene density in that region. The GSEA algorithm tests for over-representation of adjusted gene scores in a given pathway using a pre-defined score rank cutoff (in our case, the 95th and 75th percentile). The generated statistic is then compared with 10 000–1 000 000 gene sets of identical size randomly sampled from the genome to generate an empirical *P*-value for each pathway. In total, 3216 pathways from Gene Ontology, PANTHER, Ingenuity, KEGG, REACTOME and BIOCARTA were tested. Pathways were considered significant if the FDR-adjusted *P*-value (the 95th or 75th percentile) was ≤0.05.

To determine the relationship between loci associated with survival ≥90 years, we used GRAIL (http://www.broadinstitute.org/mpg/grail/) (34). In short, this program maps SNPs to genes and subsequently uses a text-mining algorithm on PubMed abstracts to determine connections between these genes. Genes from independent loci, which share informative words, receive a high GRAIL similarity score and are more likely to be functionally related. As we only had a limited number of loci with at least one SNP with a *P*-value ≤1 × 10^−5^ (*n* = 12, Table 2), we decided to perform GRAIL analysis on all loci with at least one SNP with a *P*-value ≤1 × 10^−4^ (*n* = 65).

### eQTL analysis

To determine whether rs2149954 or SNPs in LD (*r*^2^ ≥ 0.8 based on 1000 Genomes CEU Phase 1 data) influenced gene expression, we searched several eQTL databases, namely (1) the Gutenberg Heart Study database (GHS_Express) (48), which is based on expression data of monocytes; (2) the Genotype-Tissue Expression (GTEx) eQTL database (http://www.ncbi.nlm.nih.gov/gtex/GTEX2/gtex.cgi), which is based on expression data of brain (cerebellum, frontal cortex, temporal cortex and pons), liver and lymphoblastoid cell lines; (3) the GENe Expression VARiation (Genevar) database (http://www.sanger.ac.uk/resources/software/genevar/), which is based on expression data of adipose tissue, fibroblasts, T cells, skin and lymphoblastoid cell lines (49) and (4) the Blood eQTL browser (http://genenetwork.nl/bloodeqtlbrowser/) (50).

## SUPPLEMENTARY MATERIAL

Supplementary Material is available at *HMG* online.

## FUNDING

This work was supported by the Augustinus Foundation; Avera Institute for Human Genetics (AIHG); AXA Research Fund; Belfast City Hospital Trust Fund, Research and Education into Ageing-0153; Biobanking and Biomolecular Resources Research Infrastructure (BBMRI –NL, NWO 184.021.007); Biotechnology and Biological Sciences Research Council (BBSRC); Bristol-Myers Squibb; Center for Inherited Disease Research (CIDR); Centre for Medical Systems Biology (CMSB); CERA Foundation; Commissariat à L'Energie Atomique (CEA)-Centre National de Génotypage (CNG); Danish Agency for Science, Technology and Innovation (DASTI)/The Danish Council for Independent Research (DCIR, grant 11-107308); Danish National Research Foundation (DNRF); Department of Health and Social Services (Northern Ireland); DFG-Cluster of Excellence ‘Inflammation at Interfaces’; Dunhill Medical Trust (grant R124/0509); Egmont Foundation; Estonian Science Foundation (grant 7859); Estonian Government (grant SF0180142s08); European Research Council (ERC, advanced grant 230374); European Science Foundation (ESF, EU/QLRT-2001-01254); European Union's Fifth/Sixth/Seventh Framework Programmes (FP5-QLK6-CY-2001-00128, FP6-LIFESCIHEALTH-36894, FP6-LSHM-CT-2004-503270, FP7-HEALTH-2007-B-223004, FP7-HEALTH-F4-2007-201413, FP7-HEALTH-F4-2008-202047, FP7-HEALTH-2009-single-stage-242244 and FP7-HEALTH-2010-two-stage-259679); Fondation Caisse d'Epargne Rhône-Alpes Lyon CERAL (2004–2007); Genetic Association Information Network (GAIN) of the Foundation for the US National Institutes of Health (NIMH, grant MH081802); GenomEUtwin (EU/QLRT-2001-01254; QLG2-CT-2002-01254); Guy's & St Thomas' NHS Foundation Trust; Health Foundation; Heart and Lung foundation (grant 20070481); Innovation-Oriented Research Program on Genomics (SenterNovem, grant IGE05007); Institute for Ageing and Health; Institut National de la Recherche Agronomique (INRA); Institut National de la Santé et de la Recherche Médicale (INSERM); INTERREG 4A programme Syddanmark-Schleswig-K.E.R.N (with EU funds from the European Regional Development Fund); King's College London; Medical Research Council (MRC, grant G0500997 and G0601333); Ministère de l'Enseignement supérieur et de la Recherche (MESR); National Institutes of Health (NIH)/National Institute of Aging (NIA, P01AG08761, R01D0042157-01A and U01DK066134); National Institute for Health Research (NIHR) Newcastle Biomedical Research Centre; NBIC BioAssist (NWO-NBIC/BioAssist/RK/2008.024); Netherlands Consortium for Healthy Ageing (NCHA, grant 050-060-810); Netherlands Genomics Initiative (NGI); Netherlands Heart Foundation (NHF, grant 2001 D 032); Netherlands Organization for Scientific Research (NWO, MagW/ZonMW grant 904-61-090, 904-61-193, 480-04-004, 400-05-717, Spinozapremie 56-464-14192, 175.010.2005.011, 911-03-012, 985-10-002, Addiction-31160008 and Middelgroot-911-09-032); Netspar – Living longer for a good health; NHS North of Tyne (Newcastle Primary Care Trust); Pharmacy Foundation; Regione Autonoma della Sardegna; Rutgers University Cell and DNA Repository (NIMH U24 MH068457-06); Swedish Research Council (grant M-2005-1112); The Competitive Research Funding of the Tampere University Hospital and Academy of Finland; The Danish Interdisciplinary Research Council; The Health Foundation (Helsefonden); The Ministry for Higher Education; The National Program for Research Infrastructure 2007 (grant 09-063256); The March of Dimes Birth Defects Foundation; The Swedish Foundation for Strategic Research (SSF); Unilever Discover Colworth; Université Paris 13; University of Calabria; University of Tartu (grant SP1GVARENG); Velux Foundation; VU University's Institute for Health and Care Research (EMGO+) and Neuroscience Campus Amsterdam (NCA); Wellcome Trust (grant 084762, 085475 and 087436). Funding to pay the Open Access publication charges for this article was provided by IDEAL (FP7-HEALTH-2010-two-stage-259679).

## Supplementary Material

Supplementary Data
