# Supplementary material for: Genome-wide association meta-analysis of human longevity identifies a novel locus conferring survival beyond 90 years of age
Source: Hum Mol Genet. 2014 Mar 31;23(16):4420–32. doi: 10.1093/hmg/ddu139 (PMC4103672; doi:10.1093/hmg/ddu139)
Supplement: Supplementary Data [file supp_23_16_4420__index.html]

Genome-wide association meta-analysis of human longevity identifies a novel locus conferring survival beyond 90 years of age — Genome-wide association meta-analysis of human longevity identifies a novel locus conferring survival beyond 90 years of age — Supplementary Data 

# Genome-wide association meta-analysis of human longevity identifies a novel locus conferring survival beyond 90 years of age

## Supplementary Data

Supplementary Data

**Files in this Data Supplement:**

- Supplementary Material - doc file
- Summary results - txt file
- Summary Results Readme - txt file
