# Supplementary material for: Genome-wide association meta-analysis of human longevity identifies a novel locus conferring survival beyond 90 years of age
Source: Hum Mol Genet. 2014 Mar 31;23(16):4420–32. doi: 10.1093/hmg/ddu139 (PMC4103672; doi:10.1093/hmg/ddu139)
Supplement: Supplementary Data [file supp_ddu139_ddu139supp.doc]

**Description of study cohorts**

**Belfast Elderly Longitudinal Free-living Ageing STudy**

The participants were drawn from a longitudinal study of ageing-Belfast Elderly Longitudinal Free-living Ageing STudy (BELFAST) as previously described (1). Briefly, BELFAST octo/nonagenarians were recruited with General Practitioner help, from the Greater Belfast Area (population approximately 260000) and seen at home by a research officer. At recruitment, all were community-living, apparently well, independently mobile, mentally competent (Folstein score > 25/30 with 30 being highest score) (2), met the exacting SENIEUR criteria (3) and gave written consent. DNA extraction was performed by standard methods. Ethical permission was given by The Queen’s University of Belfast Ethics Committee.

**Calabria cohort**

The Calabrian sample of the present study is represented by 554 unrelated individuals (age range 20 - 108 years; median ages 74.22 ± 25.67), 209 males and 345 females. The group of long-lived subjects consists of 309 individuals (age range 85 - 108 years; median ages 95.28 ± 5.44), 107 males and 202 females. The control group consists of 245 individuals (age range 20-65 years; median ages 47.67 ± 13.65), 102 males and 143 females. All individuals were born in Calabria (Southern Italy) and their ancestry in the region had been ascertained up to the grandparent’s generation. The sample has been recruited in the frame of the ECHA project (2002 - 2004) and of the MUSA Project (2008 onward). Details on the sampling strategies have been described elsewhere (4). Phenotypic information was collected using the questionnaires available at <http://biologia.unical.it/echa/results.htm>.

**CEPH centenarian cohort**

French centenarians were recruited when they were in their 100th year or beyond. French siblings were recruited when at least two siblings fulfilling age criteria of 90 years or older were alive in a family. The mean age of centenarians and unrelated siblings was respectively 104 and 100 years (age at death or age at last contact). In total 1234 elderly were included in the study (998 centenarians and 236 unrelated members from a sibling). DNA was extracted from peripheral blood lymphocytes by standard methods (5).

**Danish longevity study I**

A total of 398 longevity cases were drawn from 4 nation-wide surveys collected at University of Southern Denmark in the years 1995 through 2007; The Study of Danish Old Sibs (DOS), the 1905 birth cohort study (6), the Danish Longitudinal Centenarian Study (DLCS) (7), and the Longitudinal Study of Ageing Danish Twins (LSADT) (8). Briefly, DOS was initiated in 2004 and included families where at least two sibs were ≥ 90 years of age at intake. The LSADT study was initiated in 1995 and includes Danish twins ≥ 70 years of age. The follow-up studies of the 1905 and DLCS cohorts were initiated in 1998 and 1995 when the participants were 92 - 93 and 100 years of age, respectively. For DOS and LSADT, one individual from each sib/twin pair was randomly selected and only among participants that had reached the age of at least 90 years (mean age 97.9 years (range 91.3 - 105.7) for DOS, and 93.5 years (range 90 - 103) for LSADT). For the 1905 cohort only participants that had become centenarians were included.

DNA from whole blood was extracted using standard methods (9).

**Danish longevity study II**

This study population was based on 4 nation-wide birth cohort surveys collected at University of Southern Denmark; the 1905 Birth Cohort Study initiated in 1998, and the 3 recent surveys of the 1910 Birth Cohort, the 1911/12 Birth Cohort, and the 1915 Birth Cohort. The 1910 and 1915 birth cohorts were collected in 2010 when participants became 100 and 95 years old, respectively, the 1911 - 1912 cohort study, is part of the international 5-Country Oldest Old Project (5-COOP) (10) conducted in 2011 and 2012 and includes individuals reaching an age of 100 years. For the 1905 and 1915 cohorts only members reaching a minimum age of 96 years were included in the study population, with no overlap of 1905 cohort participants included in The Danish longevity study I. In total, the study population includes 606 individuals with a mean age of 98.4 (range 96 - 100) years. DNA from whole blood was extracted using standard methods (9). DNA from filter cards was extracted using the Extract-N-Amp Blood PCR Kit (Sigma Aldrich, St. Louis, MO, USA) and subsequently amplified using the GenomePlex Complete Whole Genome Amplification (WGA) Kit (Sigma Aldrich, St. Louis, MO, USA).

**deCODE**

deCODE has reported the use of a comprehensive population-based computerized genealogy database to examine multigenerational relationships among those who live to the 95th percentile in Iceland (11). Results show that first degree relatives of those living to the 95th percentile were almost twice as likely to live to the 95th percentile compared with controls. In the current study longevity variants are tested for association in individuals ≥ 85 years of age (*n* = 9591) compared to those who died before 65 or are younger than 65 years of age (*n* = 56344) at the time of the study. Renewed approval for the longevity project was obtained from the The National Bioethics Committee of Iceland (99-032-V1, 2009) and The Data Protection Authority of Iceland (209020153ÞS, 2009).

**Estonian Biobank**

The Estonian Biobank is the population-based biobank of the Estonian Genome Center at the University of Tartu (www.biobank.ee; EGCUT). The entire project is conducted according to the Estonian Gene Research Act and all of the participants have signed the broad informed consent. The cohort size is up to 51535 individuals from 18 years of age and up, which closely reflects the age, sex and geographical distribution of the Estonian population. All of the subjects are recruited randomly by general practitioners and physicians in hospitals. A Computer Assisted Personal interview is filled within 1 - 2 hours at a doctor’s office, which includes personal, genealogical, educational, occupational history and lifestyle data. Anthropometric measurements, blood pressure and resting heart rate are measured during the visit. 329 female cases and 403 female controls were included in the meta-GWAS. Prospective analysis was carried out on 7012 individuals (including 1247 deceased cases).

**Genetics of Healthy Aging Study**

The individuals investigated in this study participate in Genetics of Healthy Aging (GEHA) Study (12). Families participating in the GEHA study have at least two siblings meeting four inclusion criteria: (i) participants are at least 90 years old, (ii) participants have at least one living brother or sister who fulfills the first criterion and is willing to participate, (iii) the nonagenarian sibship has an identical mother and father, and (iv) the parents of the nonagenarian sibship are European and Caucasian. In total, 2249 sibships have been recruited. From each pair the eldest siblings from the United Kingdom, Netherlands, Denmark, France, and Bologna, and GEHA controls from France and Bologna were genotyped. In accordance with the Declaration of Helsinki, written informed consent was obtained from all participants prior to entering the study. Good clinical practice guidelines were maintained. The study protocol was approved by the local medical ethical committees of the 11 participating countries before the start of the study. In this study, analyses were performed separately for each country.

**German longevity study**

The long-lived sample comprised 1492 unrelated individuals of German ancestry between 95 and 110 years of age at the time of recruitment (mean age 98.9 years). About 47% (*n* = 698) of them were centenarians (mean age 101.3 years). The sex ratio in the entire sample was 73% females vs. 27% males. Long-lived cases were recruited as previously described (13). The control sample consisted of 1529 unrelated German individuals aged between 18 and 64 years (mean age 44 years) and showed a sex ratio of 53% females vs. 47% males. Control subjects were obtained from the PopGen biobank (14). DNA from blood samples was extracted using standard methods. Approval for the project was received from the Ethics Committee of the University Hospital Schleswig-Holstein, Campus Kiel.

**Genomics of extremely Overweight Young Adults**

Controls for the Danish longevity studies were derived from the Danish genome-wide association study GOYA (Genomics of extremely Overweight Young Adults) (15). In this study, female controls came initially from the Danish National Birth Cohort (1996 - 2002) where they were drawn as a random cohort sample from 67863 women who gave birth to a live born infant and provided a blood sample during pregnancy. Genotyping was successful in 2021 women. Similarly, the male controls were a random cohort sample, initially based on one in every hundred of 362000 Caucasian men, drafted in Copenhagen from 1943 - 77 (*n* = 3601). In 1992 - 94, half of the random cohort still living in the Copenhagen area were invited to a follow-up where blood samples were taken. Genotyping was successful in 796 men. For the case-control analyses in this study, the samples from GOYA were divided into three separate groups that were used as controls for the Danish longevity studies (GOYA I and II) and GEHA Danish (GOYA III).

**Leiden 85-plus study**

In the Leiden 85-plus study, two prospective population-based cohorts were recruited from inhabitants of Leiden (16,17). Between 1987 and 1989, 673 subjects aged 85 years and older were enrolled in a prospective study (Cohort I). Between 1997 and 1999, 563 individuals were enrolled in the month of their 85th birthday with follow-up (Cohort II). Subjects were visited at their home, and there were no exclusion criteria related to health. DNA was available from the combined cohorts consisting of 1208 individuals aged 85 years and older. For the case-control analyses in this study, the samples from the Leiden 85-plus study were divided into two separate groups, one consisting of individuals from Cohort II for which GWAS data was available (Leiden 85-plus study I) and the other one consisting of the remaining individuals from Cohort I and II (Leiden 85-plus study II).

**Leiden Longevity Study**

For the Leiden Longevity Study (LLS), long-lived siblings of European descent were recruited together with their offspring and the partners of the offspring. Families were included if at least two long-lived siblings were alive and fulfilled the age criterion of 89 years or older for men and 91 years or older for women, representing < 0.5% of the Dutch population in 2001 (18). In total, 944 long-lived proband siblings from 421 families were included with a mean age of 94 years (range 89 – 104), together with 1671 of their offspring (61 years, 39 – 81) and 744 partners thereof (60 years, 36 – 79). DNA from the LLS was extracted from samples at baseline using conventional methods (19). For the case-control analyses in this study, the LLS nonagenarians were used as cases (LLS I) and the LLS partners were divided into two separate groups that were used as controls for the GEHA Dutch (LLS II) and Leiden 85-plus study I (LLS III).

**Newcastle 85+ Study**

The longitudinal Newcastle 85+ Study recruited in 2006-7 a total cohort of 1042 individuals all born in 1921, of whom 852 underwent full assessment (the remainder agreeing to review of medical records) (20). Given the near universal coverage of the NHS among people of this age and in this region, effectively all those in the age group were approached to participate, including those in care homes and with disabilities and/or cognitive impairment. A good level of recruitment was achieved with a high degree of representativeness in the study sample. DNA was extracted at baseline using conventional methods for 765 individuals for whom adequate blood samples were available. Genotype data were obtained 710 individuals and following quality control checks 642 individuals were retained and contributed to the final analysis. 432 individuals remained alive at age 90.

**Netherlands Twin Registry**

From the Netherlands Twin Registry (NTR), 2021 unrelated participants of European descent for whom DNA was available were selected as control samples (21). The substructure in the NTR has been reported before (22), and in this study, we included samples aged between 15 and 65 years at the time of blood sampling, without known family relations. For the case-control analyses in this study, the samples from NTR were divided into two separate groups that were used as controls for BELFAST (NTR I) and Leiden 85-plus study II (NTR II).

**PROspective Study of Pravastatin in the Elderly at Risk**

All data come from the PROspective Study of Pravastatin in the Elderly at Risk (PROSPER). A detailed description of the study has been published elsewhere (23,24). PROSPER was a prospective multicenter randomized placebo-controlled trial to assess whether treatment with pravastatin diminishes the risk of major vascular events in elderly. Between December 1997 and May 1999, we screened and enrolled subjects in Scotland (Glasgow), Ireland (Cork), and the Netherlands (Leiden). Men and women aged 70-82 years were recruited if they had pre-existing vascular disease or increased risk of such disease because of smoking, hypertension, or diabetes. A total number of 5804 individuals were randomly assigned to pravastatin or placebo. DNA was available for genotyping in 5763 individuals (25).

**Rotterdam Study**

The Rotterdam study is a population-based cohort study that investigates the occurrence and determinants of diseases in the elderly (26). Baseline examinations, including a detailed questionnaire, physical examination and blood collection, were conducted between 1990 and 1993. The Medical Ethics Committee at Erasmus Medical Center approved the study protocol. In this study, analyses were performed separately for the first (Rotterdam Study I) and second (Rotterdam Study II) cohort.

**Supplementation in Vitamins and Mineral Antioxidants**

Controls for CEPH Centenarians were selected in a population-based sample of French subjects that had participated in the Supplementation in Vitamins and Mineral Antioxidants (SU.VI.MAX) study (27).

**TwinGene**

The participants were obtained from the TwinGene project which is a population-based study of Swedish twins born between 1911 and 1958. The study was collected between 2004 and 2008 and both twins within a pair had to be alive at time of testing to be eligible to participate. The study participants had previously taken part in a telephone-based interview called Screening Across the Lifespan (SALT), which took place between 1998 and 2002. Those who had donated DNA for previous Swedish Twin Registry studies, who had a record of hepatitis, or who had declined participation in further studies were excluded from the study. In total, 12647 individuals participated and 11991 individuals donated blood to the study. Sampling and clinical blood test procedures have been described elsewhere (28). TwinGene has been linked to the Swedish national patient register and Cause of death register. The study was approved by the regional ethical review board at Karolinska Institute and all participants gave informed consent.

**TwinsUK**

UK population-based control data were obtained from the TwinsUK resource (http://www.twinsuk.ac.uk), an adult twin registry comprising 12000 (predominantly female) twins. Genotype data for 3512 twin individuals were obtained from the Department of Twin Research and Genetic Epidemiology at King’s College, London. In the current study we used 250 randomly selected individuals (after excluding population outliers) below 65 years of age at inclusion.

**Wellcome Trust Case Control Consortium 2**

UK population-based control data were obtained from the Wellcome Trust Case Control Consortium 2 (WTCCC2) (https://www.wtccc.org.uk/ccc2) and comprise individuals from two collections, the 1958 Birth Cohort (58C) and a National Blood Donor Collection (NBS). In the current study we used all individuals below 65 years of age at inclusion (*n* = 5114).

**Supplemental acknowledgements**

**BELFAST**

The Belfast Elderly Longitudinal Free-living Aging STudy (BELFAST) was funded in part by a grant from the Department of Health and Social Services (Northern Ireland) and Belfast City Hospital Trust Fund, Research and Education into Ageing-0153.

**Calabria cohort**

The ECHA project received funds from EU, under the 5th FP (ECHA project; Contract

QLK6-CY-2001-00128). MUSA study is routinely sustained with funds of the University of Calabria.

**CEPH centenarian cohort**

The CEPH centenarian cohort study was funded by the ‘‘Ministère de l’Enseignement supérieur et de la Recherche’’ and the Commissariat à L'Energie Atomique-Centre National de Génotypage. The authors thank the CEPH Biological Resource Centre and the CNG Genotyping lab for technical assistance.

**Danish longevity studies**

The Danish longevity studies have received funding from The National Program for Research Infrastructure 2007 (grant no. 09-063256) from the Danish Agency for Science Technology and Innovation, the Velux Foundation, the US National Institute of Health (P01 AG08761), and from the Danish Agency for Science, Technology and Innovation/The Danish Council for Independent Research (grant no. 11-107308), and **The Danish Interdisciplinary Research Council. The 5-COOP project (5-Country Oldest Old Project) is funded by the CERA Foundation (Lyon) and the AXA Research Fund, Paris, and The Health Foundation (Helsefonden), Copenhagen, Denmark.** This study was supported by grants from the European Union's Seventh Framework Programme (FP7/2007-2011) under grant agreement n° 259679, and the INTERREG 4 A programme Syddanmark-Schleswig-K.E.R.N. (by EU funds from the European Regional Development Fund).

**EGCUT**

EGCUT received financing from FP6 grant #36894, targeted financing from Estonian Government grant SF0180142s08, Estonian Science Foundation (grant no. 7859) and University of Tartu (SP1GVARENG). We acknowledge EGCUT technical personnel, especially Mr V. Soo and S. Smit. Data analyzes were carried out in part in the High Performance Computing Center of University of Tartu.

**GEHA**

The work described in this study was funded mainly by the EU GEHA Project contract no. LSHM-CT-2004-503-270. The work has additionally been supported by the following programs and agencies: The Competitive Research Funding of the Tampere University Hospital and Academy of Finland (Tampere); United States National Institute of Aging (PO1-AG08761) (Odense); The Innovation Oriented Research Program on Genomics (Senter-Novem IGE05007), the Centre for Medical Systems Biology (CMSB), and the National Institute for Healthy Ageing (NCHA 05060810), all in the framework of the Netherlands Genomics Initiative (NGI)/Netherlands Organisation for Scientific Research (NWO) (Leiden); The Institute for Ageing and Health and the UK NIHR Biomedical Research Centre for Ageing and Age-related disease award to the Newcastle-upon-Tyne Foundation Hospitals NHS Trust (Newcastle); Fondation Caisse d’Epargne Rh^one-Alpes Lyon CERAL (2004–2007) (Montpellier); Regione Autonoma della Sardegna (Sassari), European Union’s Seventh Framework Programme (FP7/2007-2011) IDEAL-ageing under grant agreement no. 259679.

**GOYA**

The GOYA study was conducted as part of the activities of the Danish Obesity Research Centre (DanORC, www.danorc.dk) and The MRC centre for Causal Analyses in Translational Epidemiology (MRC CAiTE). The genotyping for GOYA was funded by the Wellcome Trust (WT 084762). GOYA is a nested study within The Danish National Birth Cohort which was established with major funding from the Danish National Research Foundation. Additional support for this cohort has been obtained from the Pharmacy Foundation, the Egmont Foundation, The March of Dimes Birth Defects Foundation, the Augustinus Foundation, and the Health Foundation.

**German longevity study**

The German longevity study at the Institute of Clinical Molecular Biology, Christian-Albrechts-University of Kiel, Germany received funding from the DFG-Cluster of Excellence "Inflammation at Interfaces", the INTERREG 4A programme Syddanmark–Schleswig–K.E.R.N (with EU funds from the European Regional Development Fund) and the EU project RESOLVE (FP7-HEALTH-F4-2008-202047).

**LLS**

The Leiden Longevity Study has received funding from the European Union's Seventh Framework Programme (FP7/2007-2011) under grant agreement n° 259679. This study was supported by a grant from the Innovation-Oriented Research Program on Genomics (SenterNovem IGE05007), the Centre for Medical Systems Biology, and the Netherlands Consortium for Healthy Ageing (grant 050-060-810), all in the framework of the Netherlands Genomics Initiative, Netherlands Organization for Scientific Research (NWO), Unilever Colworth and by BBMRI-NL, a Research Infrastructure financed by the Dutch government (NWO 184.021.007).

**Newcastle 85+ Study**

The Newcastle 85+ Study has received funding from the Medical Research Council (G0500997); the Dunhill Medical Trust (R124/0509); the Medical Research Council and Unilever Discover Colworth (G0601333) and NHS North of Tyne (Newcastle Primary Care Trust). This study was additionally funded from the EU Network of Excellence LIFESPAN and from the Biotechnology and Biological Sciences Research Council support for Newcastle University’s Centre for Integrated Systems Biology of Ageing and Nutrition. The research was further supported by the National Institute for Health Research (NIHR) Newcastle Biomedical Research Centre based at Newcastle upon Tyne Hospitals NHS Foundation Trust and Newcastle University. Statistical analysis of genotype data from the Newcastle 85+ Study was supported by funding from the Wellcome Trust (grant number 087436).

**NTR**

Funding was obtained from the Netherlands Organization for Scientific Research (NWO: MagW/ZonMW grants 904-61-090, 985-10-002, 904-61-193,480-04-004, 400-05-717, Addiction-31160008 Middelgroot-911-09-032, Spinozapremie 56-464-14192), Center for Medical Systems Biology (CSMB, NWO Genomics), NBIC/BioAssist/RK(2008.024), Biobanking and Biomolecular Resources Research Infrastructure (BBMRI –NL, 184.021.007), the VU University’s Institute for Health and Care Research (EMGO+ ) and Neuroscience Campus Amsterdam (NCA), the European Science Foundation (ESF, EU/QLRT-2001-01254), the European Community's Seventh Framework Program (FP7/2007-2013), ENGAGE (HEALTH-F4-2007-201413); the European Science Council (ERC Advanced, 230374), Rutgers University Cell and DNA Repository (NIMH U24 MH068457-06), the Avera Institute, Sioux Falls, South Dakota (USA) and the National Institutes of Health (NIH, R01D0042157-01A). Part of the genotyping and analyses were funded by the Genetic Association Information Network (GAIN) of the Foundation for the US National Institutes of Health, the (NIMH, MH081802).

**PROSPER**

The PROSPER study was supported by an investigator initiated grant obtained from Bristol-Myers Squibb. Prof. Dr. J. W. Jukema is an Established Clinical Investigator of the Netherlands Heart Foundation (grant 2001 D 032). Support for genotyping was provided by the seventh framework program of the European commission (grant 223004) and by the Netherlands Genomics Initiative (Netherlands Consortium for Healthy Aging grant 050-060-810).

**Rotterdam Study**

The Rotterdam Study is supported by Netherlands Genomics Initiative/Netherlands Consortium for Healthy Aging (050-060-810); Netherlands Organisation for Scientific Research (NWO) (904-61-090, 904-61-193, 480-04-004, 400-05-717, SPI 56-464-1419, 175.010.2005.011 and 911-03-012); Netspar – Living longer for a good health; Centre for Medical Systems Biology (CMSB); European Community’s Seventh Framework Programme (CHANCES consortium: 242244).

**SU.VI.MAX**

The SU.VI.MAX cohort team was funded by the French Institut National de la Santé et de la Recherche Médicale, the Institut National de la Recherche Agronomique, the Université Paris 13 and the Commissariat à L'Energie Atomique-Centre National de Génotypage.

**TwinGene**

The Ministry for Higher Education, the Swedish Research Council (M-2005-1112), GenomEUtwin (EU/QLRT-2001-01254; QLG2-CT-2002-01254), NIH DK U01-066134, The Swedish Foundation for Strategic Research (SSF). Heart and Lung foundation no. 20070481.

**TwinsUK**

The study was funded by the Wellcome Trust; European Community’s Seventh Framework Programme (FP7/2007-2013). The study also receives support from the Dept of Health via the National Institute for Health Research (NIHR) comprehensive Biomedical Research Centre award to Guy's & St Thomas' NHS Foundation Trust in partnership with King's College London. SNP Genotyping was performed by The Wellcome Trust Sanger Institute and National Eye Institute via NIH/CIDR.

**WTCCC2**

This study made use of data generated by the Wellcome Trust Case-Control Consortium 2 (WTCCC2). A full list of the investigators who contributed to the generation of the data is available from http://www.wtccc.org.uk. Funding for the WTCCC2 project was provided by the Wellcome Trust under award 085475.

**Members of the Genetics of Healthy Aging (GEHA) consortium:**

**Catholic University of Louvain, Louvain, Belgium**

Christine Guilbault, Christel Lecomte, Gisèle Vandervelpen and Michel Poulain

**Facultés Universitaires Notre-Dame de la Paix, Namur, Belgium**

Florence Chainiaux, Thierry Pascal and Olivier Toussaint

**Eppendorf Array Technologies, Namur, Belgium**

José Remacle

**Chinese Academy of Sciences, Beijing, China**

Lars Bolund

**University of Southern Denmark, Odense, Denmark**

Susanne Andersen, Frans Bødker, Gitte Bay Christensen, Svend Erik Christiansen, Susanne Kløjgaard, Susanne Knudsen, Lene Nielsen, Kirsten M. Rasmussen, Helene Rønne, Axel Skytthe, Jytte Skaaning, Kaare Christensen and Bernard Jeune

**University of Aarhus, Aarhus, Denmark**

Peter Kristensen

**The National Institute for Health and Welfare, Helsinki, Finland**

Minttu Jussila, Helena Knuuttila, Paivi Laiho, Markus Perola, Outi Repola Törnwall, Elisabeth Widén and Leena Peltonen

**Tampere School of Public Health, Tampere, Finland**

Marika Ervasti, Maija Heinünen, Suija Hirviniemi, Marja Jylha, Anno-Maria Korhonen, Kirsi Māempāā, Eeva Orsali, Jaana Repo, Katariina Saatela, Anne Tuikkala, Jaana Tuovinen, Pia Turkvata, Karoliina Verkkonen, Kaisa Yli-Hukkala, Anniina Aalto and Antti Hervonen

***INSERM, Montpellier, France***

Frédéric Balard, Marie-Hélène Castaladi, Aurore Clavel, Franck Dewit, Joëlle Faurobert, Angélique Frapsauce, Bruno Granger, Marjorie Marais, Béatrice Martin, Michelle Phelipot, Isabelle Romieu, Nicolas Snoek and Jean Marie Robine

**Fondation Jean Dausset-CEPH, Paris, France**

Hélène Blanché and Mark Lathrop

**Christian-Albrechts-University, Kiel, Germany**

Sonja Börm, Kerstin Milbradt, Almut Nebel, Susanna Nikolaus, Ulrike Peters, Huberta von Eller-Eberstein and Stefan Schreiber

**Max Planck Institute for Demographic Research, Rostock, Germany**

Jutta Gampe and James W. Vaupel

**National Hellenic Research Foundation, Athens, Greece**

Yanna Ioannou, Aggeliki Skouteri, Katerina Theodorelou, Christos Tzavelas, Konstantinos Voutetakis and Stathis Gonos

**University of Bologna, Bologna, Italy**

Laura Celani, Elisa Cevenini, Maria Giustina Palmas, Maria Panagiota Panourgia, Elisa Pini, Maria Scurti, Federica Sevini, Silvana Valensin and Claudio Franceschi

**Istituto Superiore di Sanità, Rome, Italy**

Rodolfo Cotichini, Cristina D’ippolito, Raniero Facchini, Daniela Ferrari, Nicoletta Lo Curatolo, Rosalba Masciulli, Silvia Meucci, Miriam Salemi, Francesca Tango, Virgilia Toccaceli and Antonia Stazi

**University of Calabria, Calabria, Italy**

Maurizio Berardelli, Serena Dato, Francesco De Rango, Vincenzo Mari, Cinzia Martino, Adriana Vallone, Giovanna De Benedictis and Giuseppe Passarino

**University of Sassari, Sardinia, Italy**

Ciriaco Carru, Andrea Marchisio, Gianni Pes, Sabrina Purcheddu, Egidio Riciardi and Luca Deiana

**Research & Innovation Soc.Coop.R.L., Padova, Italy**

Alberta Leon

**Fondazione Intituto FIRC di Oncologia Molecolare, Milano, Italy**

Pier Giuseppe Pelicci

**Italian National Research Center on Aging, Ancona, Italy**

Liana Spazzafumo

**Leiden University Medical Center, Leiden, The Netherlands**

Marian Beekman, Ellen Bemer, Gerard-Jan Blauw, Anton J.M. de Craen, Myriam de Groot-Nys, Liesbeth W. de Man, Marijke Frölich, Inge G.J. Mooijekind, Trudi van Boxsel, Ruud van der Breggen, Meriam G.H.F van der Star, Rudi G.J. Westendorp and P. Eline Slagboom

**Nencki Istitute of Experimental Biology, Warszawa, Poland**

Katarzyna Broczek, Beata Frąckowiak, Dorota Janiszewska, Małgorzata Kupisz-Urbańska, Renata Podkówka-Sieczka and Ewa Sikora

**Institute of Gerontology, Kiev, Ukraine**

Victor Kcholin, Vitaliy Kirik, Khachik Muradian, Kira Poletayeva, Alexander Polyakov, Irina Polyakova, Natalya Prokopenko, Pavel Slynchak, Tatyana Stelmakch, Elena Tomarevskaya, Alina Ustimenko, Alexander Vaiserman, Galina Vavrik and Vladyslav Bezrukov

**Queens University of Belfast, Belfast, UK**

Susan McNerlan, Anne Murphy and Irene Maeve Rea

**Newcastle University, Newcastle, UK**

Joanna C. Collerton, Karen Davies, Joan Hughes and Thomas B.L. Kirkwood

**Figure S1.** Quantile-quantile plots. Quantile-quantile plots of the expected versus (unadjusted) observed *Χ^2^* values for the discovery phase analysis of cases aged ≥ 85 years (A) and ≥ 90 years (B). The shaded region represents the 95% confidence band.

**Figure S2.** Regional association plot for the chromosome 19q13.32 locus after conditional analysis for rs4420638. Results of the discovery phase analysis of chromosome 19q13.32 after conditional analysis in cases aged ≥ 85 years (generated using LocusZoom (<http://csg.sph.umich.edu/locuszoom/>) (29)). The color of the SNPs is based on the LD with the lead SNP (shown in purple). The blue peaks represent the recombination rates based on HapMap Phase I + II CEU release 22 (hg18/build36) and the RefSeq genes in the region are shown in the lower panel.

**
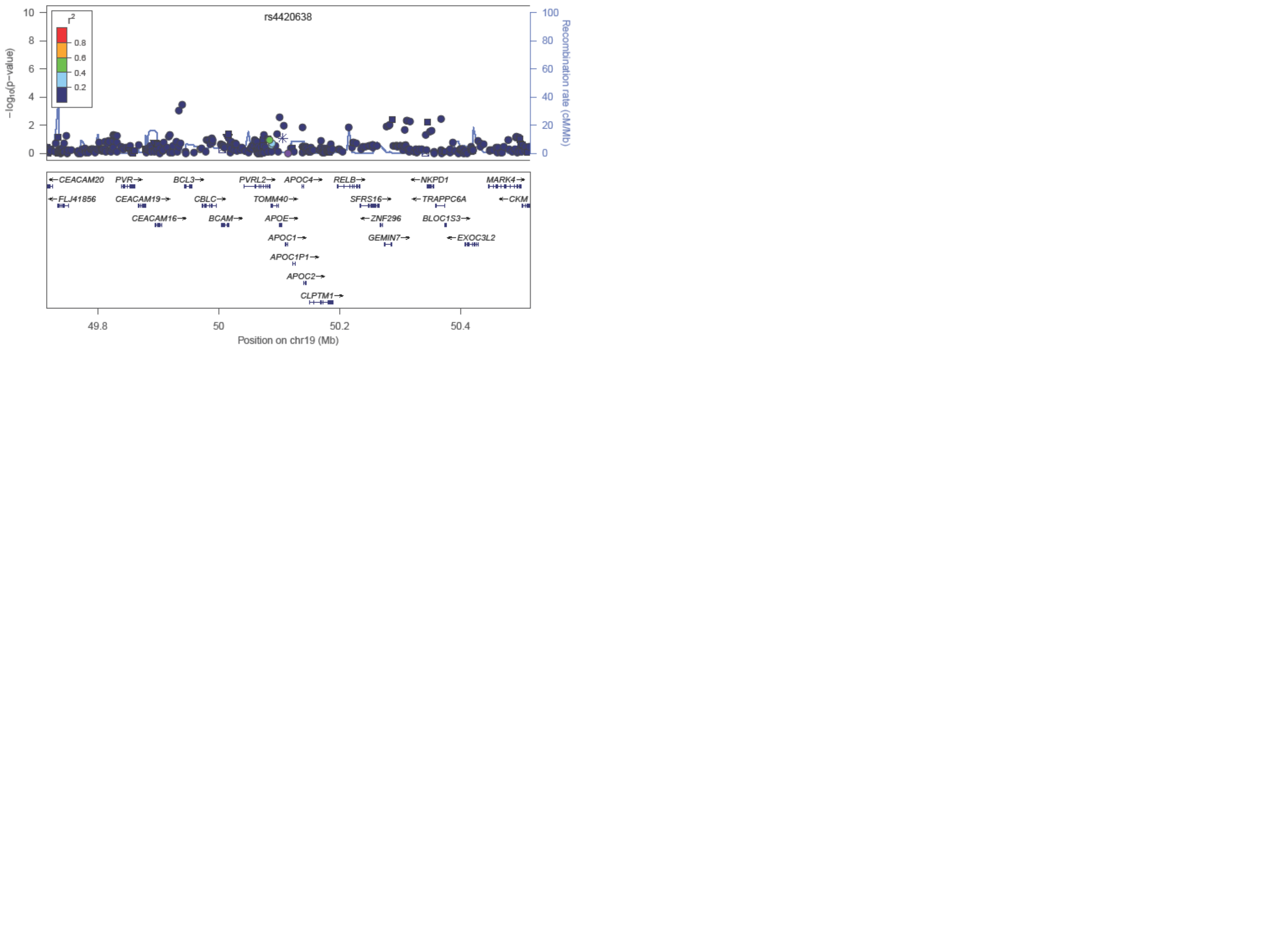
**

**Figure S3.** Graphical representation of GRAIL connections. Results of the GRAIL analysis using the loci with at least one SNP with a *P* ≤ 1 x 10^-4^ in the discovery analysis of cases aged ≥ 90 years (*n* = 65). The thickness of the red line represents the strength of the literature-based connection.


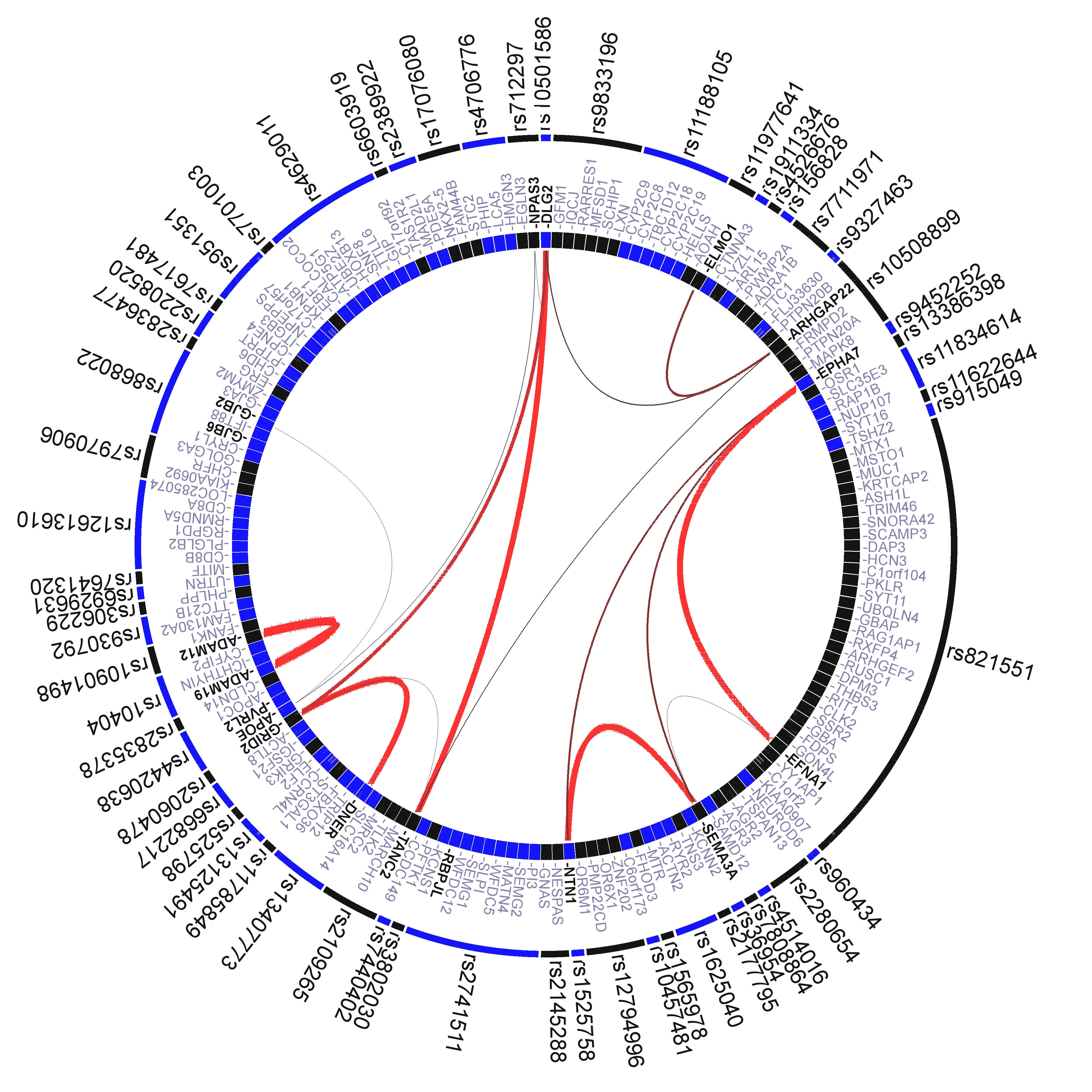


**Figure S4.** Quantile-quantile plot of the expected versus (unadjusted) observed *Χ^2^* values for the 281 SNPs from the signature of Sebastiani et al. (30) in the analysis of cases aged ≥ 90 years. The shaded region represents the 95% confidence band. The red line indicates the threshold for significance after adjustment for multiple testing (*P* < 1.78 x 10^-4^ (0.05/281)).


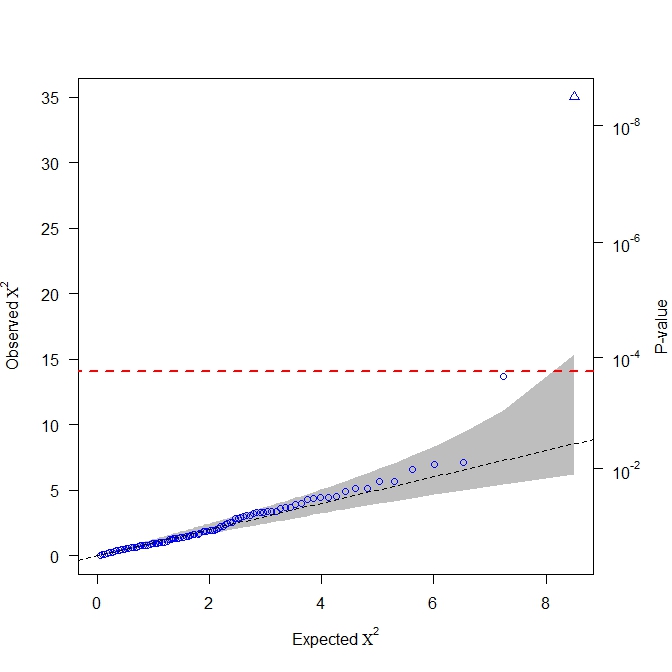


**Table S1.** Cohort demographics of the studies of European descent included in the discovery and replication phase.

| **Study** | **Phase** | **Group** | **Cohort** | **Country** | ***n*** | **Mean Age (SD)** | **Age Range** | **Men** |
| --- | --- | --- | --- | --- | --- | --- | --- | --- |
| CEPH centenarian cohort | Discovery | Cases | CEPH centenarian cohort | France | 1234 (1234) | 102.4 (3.0) | 91 - 114+ | 230 (18.6%) |
|  |  | Controls** | SU.VI.MAX | France | 831 | 49.9 (6.2) | 35 - 62 | 340 (40.9%) |
| Danish longevity study I | Discovery | Cases | Danish longevity study I | Denmark | 398 (398) | 98.5 (4.5) | 90 - 109 | 120 (30.2%) |
|  |  | Controls** | GOYA I | Denmark | 900 | 26.5 (5.6) | 18 - 42 | 260 (28.9%) |
| EGCUT | Discovery | Cases | EGCUT  EGCUT | Estonia  Estonia | 329 (NA) | 88.4 (3.6) | 85 - 105 | 0 (0%) |
|  |  | Controls |  |  | 403 | 41.5 (13.8) | 18 - 65 | 0 (0%) |
| GEHA Danish | Discovery | Cases | GEHA Study | Denmark | 442 (442) | 93.8 (2.1) | 90 - 103 | 142 (31.8%) |
|  |  | Controls** | GOYA II | Denmark | 900 | 26.6 (5.5) | 18 - 44 | 260 (28.9%) |
| GEHA Dutch | Discovery | Cases | GEHA Study | Netherlands | 197 (197) | 94.2 (2.5) | 90 - 103 | 65 (33.0%) |
|  |  | Controls** | LLS II | Netherlands | 219 | 55.5 (5.6) | 30 - 65 | 83 (37.9%) |
| GEHA French* | Discovery | Cases | GEHA Study  GEHA Study | France  France | 295 (295) | 94.6 (2.6) | 90 - 105 | 85 (28.8%) |
|  |  | Controls |  |  | 354 | 63.4 (6.8) | 50 - 75 | 124 (35,0%) |
| GEHA Italy* | Discovery | Cases | GEHA Study  GEHA Study | Italy  Italy | 182 (182) | 95.7 (2.7) | 92 - 104 | 51 (28.0%) |
|  |  | Controls |  |  | 184 | 62.6 (6.0) | 50 - 75 | 51 (27.7%) |
| GEHA UK | Discovery | Cases | GEHA Study | UK | 112 (112) | 94.5 (2.8) | 90 - 103 | 28 (25.0%) |
|  |  | Controls** | TwinsUK | UK | 250 | 44.5 (11.6) | 18 - 64 | 125 (50.0%) |
| Leiden 85-plus study I | Discovery | Cases | Leiden 85-plus study I | Netherlands | 316 (245) | 92.7 (2.9) | 88 - 99 | 89 (28.2%) |
|  |  | Controls** | LLS III | Netherlands | 357 | 56.9 (5.8) | 32 - 65 | 126 (35.3%) |
| LLS | Discovery | Cases | LLS I | Netherlands | 934 (928) | 93.4 (2.7) | 89 - 103 | 350 (37.5%) |
|  |  | Controls** | Rotterdam Study I+II | Netherlands | 1670 | 57.7 (1.4) | 55 - 60 | 745 (44.6%) |
| Newcastle 85+ Study | Discovery | Cases | Newcastle 85+ Study | UK | 642 (381) | 85.0 (NA) | NA | 253 (39.4%) |
|  |  | Controls** | WTCCC2 (NBS+58C) | UK | 5114 | NBS: 41.0 (12.0) ; 58C: < 55 (NA) | NBS: 15 - 65 ; 58C: NA | 2,577 (50.4%) |
| Rotterdam Study I | Discovery | Cases | Rotterdam Study I  Rotterdam Study I | Netherlands  Netherlands | 2043 (892) | 90.2 (3.9) | 85 - 106 | 570 (27.9%) |
|  |  | Controls |  |  | 1219 | 62.5 (1.4) | 60 - 65 | 537 (44.1%) |
| Rotterdam Study II | Discovery | Cases | Rotterdam Study II  Rotterdam Study II | Netherlands  Netherlands | 172 (NA) | 89.4 (3.3) | 85 - 103 | 62 (36.0%) |
|  |  | Controls |  |  | 606 | 62.1 (1.3) | 60 - 65 | 268 (44.2%) |
| TwinGene | Discovery | Cases | TwinGene  TwinGene | Sweden  Sweden | 433 (100) | 87.8 (2.7) | 85 - 98 | 200 (46.2%) |
|  |  | Controls |  |  | 3114 | 59.7 (3.7) | 52 - 64 | 1359 (43.7%) |
| BELFAST | Replication | Cases | BELFAST | Northern Ireland | 206 (152) | 92.3 (4.5) | 85 - 104 | 55 (26.7%) |
|  |  | Controls | NTR I | Netherlands | 500 | 34.6 (10.2) | 18 - 64 | 136 (27.2%) |
| Calabria cohort | Replication | Cases | Calabria cohort  Calabria cohort | Italy  Italy | 309 (249) | 95.3 (5.4) | 85 - 108 | 107 (34.6%) |
|  |  | Controls |  |  | 245 | 47.7 (13.7) | 20 - 65 | 102 (41.6%) |
| Danish longevity study II | Replication | Cases | Danish longevity study II | Denmark | 606 (606) | 98.4 (2.0) | 96 - 100 | 125 (20.6%) |
|  |  | Controls** | GOYA III | Denmark | 1017 | 26.5 (5.4) | 16 - 42 | 276 (27.1%) |
| deCODE | Replication | Cases | deCODE  deCODE | Iceland  Iceland | 9591 (4272) | 89.7 (4.0) | 85 - 109 | 3,838 (40.0%) |
|  |  | Controls |  |  | 56344 | 46.7 (12.4) | 4 - 64 | 24,539 (43.6%) |
| German longevity study | Replication | Cases | German longevity study  German longevity study | Germany  Germany | 1492 (1492) | 98.9 (2.7) | 95 - 110 | 409 (27.4%) |
|  |  | Controls |  |  | 1529 | 44.0 (12.5) | 18 - 64 | 723 (47.3%) |
| Leiden 85-plus study II | Replication | Cases | Leiden 85-plus study II | Netherlands | 856 (559) | 92.4 (4.5) | 85 - 113 | 274 (32.0%) |
|  |  | Controls** | NTR II | Netherlands | 1521 | 34.2 (10.0) | 15 - 65 | 582 (38.3%) |

The columns *n, Mean Age, Age Range* and *Men* were based on the individuals above 85 years of age (cases) or below 65 years of age (controls). The number between brackets in the column *n* represents the number of individuals above 90 years of age. *For these studies geographically matched controls between 50 - 75 years were recruited and used for the analysis. **Controls were matched to cases based on country of origin.

**Table S2.** Details of genotyping, quality control and imputation of the studies of European descent included in the discovery and replication phase.

|  |  |  | **Genotyping** | | | | | **Imputation** | |  |  |
| --- | --- | --- | --- | --- | --- | --- | --- | --- | --- | --- | --- |
| **Study** | **Phase** | **Group** | **Cohort** | **Platform** | **Call rate** | **MAF** | **HWE *P*** | **Genotyped SNPs** | **Software** | **SNPs after QC** | **Lambda (λ_gc_)** |
| CEPH centenarian cohort | Discovery | Cases | CEPH centenarian cohort | Illumina Human610/Illumina Human660W | > 95% | > 1% | > 10^-4^ | 495252 | MACH | 2429449 | 1.02 |
|  |  | Controls | SU.VI.MAX |  |  |  |  |  |  |  |  |
| Danish longevity study I | Discovery | Cases | Danish longevity study I | Illumina OmniExpress | > 95% | > 1% | > 10^-4^ | 311227 | IMPUTE | 2388242 | 1.00 |
|  |  | Controls | GOYA I | Illumina Human610 |  |  |  |  |  |  |  |
| EGCUT | Discovery | Cases | EGCUT  EGCUT | Illumina OmniExpress | > 95% | > 1% | > 10^-6^ | 611535 | IMPUTE | 2454842 | 1.01 |
|  |  | Controls |  |  |  |  |  |  |  |  |  |
| GEHA Danish | Discovery | Cases | GEHA Study | Illumina Human660W | > 95% | > 1% | > 10^-4^ | 506293 | IMPUTE | 2418726 | 1.02 |
|  |  | Controls | GOYA II | Illumina Human610 |  |  |  |  |  |  |  |
| GEHA Dutch | Discovery | Cases | GEHA Study | Illumina Human660W | > 95% | > 5% | > 10^-4^ | 471236 | IMPUTE | 2131448 | 1.03 |
|  |  | Controls | LLS II | Illumina Human660W |  |  |  |  |  |  |  |
| GEHA French | Discovery | Cases | GEHA Study  GEHA Study | Illumina Human660W | > 95% | > 1% | > 10^-4^ | 300436 | IMPUTE | 2388145 | 1.01 |
|  |  | Controls |  | Illumina OmniExpress |  |  |  |  |  |  |  |
| GEHA Italy | Discovery | Cases | GEHA Study  GEHA Study | Illumina OmniExpress | > 95% | > 5% | > 10^-4^ | 573603 | IMPUTE | 2174532 | 0.99 |
|  |  | Controls |  | Illumina OmniExpress |  |  |  |  |  |  |  |
| GEHA UK | Discovery | Cases | GEHA Study | Illumina Human660W | > 95% | > 1% | > 10^-4^ | 510608 | IMPUTE | 2116844 | 0.97 |
|  |  | Controls | TwinsUK | Illumina Human610 |  |  |  |  |  |  |  |
| Leiden 85-plus study I | Discovery | Cases | Leiden 85-plus study I | Illumina OmniExpress | > 95% | > 1% | > 10^-4^ | 603301 | IMPUTE | 2460360 | 1.04 |
|  |  | Controls | LLS III | Illumina OmniExpress |  |  |  |  |  |  |  |
| LLS | Discovery | Cases | LLS I | Illumina Human660W/Illumina OmniExpress | > 95% | > 1% | > 10^-4^ | 288635 | IMPUTE | 2414730 | 1.03 |
|  |  | Controls | Rotterdam Study I+II | Illumina Infinium II HumanHap 550K/Illumina Infinium II HumanHap550-Duo |  |  |  |  |  |  |  |
| Newcastle 85+ Study | Discovery | Cases | Newcastle 85+ Study | Illumina OmniExpress | > 95% | > 1% | > 10^-4^ | 459956 | IMPUTE | 2445006 | 1.08 |
|  |  | Controls | WTCCC2 (NBS+58C) | Illumina 1.2M |  |  |  |  |  |  |  |
| Rotterdam Study I | Discovery | Cases | Rotterdam Study I  Rotterdam Study I | Illumina Infinium II HumanHap 550K | > 98% | > 1% | > 10^-6^ | 491875 | MACH | 2428937 | 1.02 |
|  |  | Controls |  |  |  |  |  |  |  |  |  |
| Rotterdam Study II | Discovery | Cases | Rotterdam Study II  Rotterdam Study II | Illumina Infinium II HumanHap550-Duo | > 98% | > 1% | > 10^-6^ | 466389 | MACH | 2117565 | 0.97 |
|  |  | Controls |  |  |  |  |  |  |  |  |  |
| TwinGene | Discovery | Cases | TwinGene  TwinGene | Illumina OmniExpress | > 97% | > 1% | > 10^-7^ | 628667 | IMPUTE | 2470389 | 1.01 |
|  |  | Controls |  |  |  |  |  |  |  |  |  |
| BELFAST | Replication | Cases | BELFAST | Sequenom |  |  |  | 15 |  | 13 |  |
|  |  | Controls | NTR I |  |  |  |  |  |  |  |  |
| Calabria cohort | Replication | Cases | Calabria cohort  Calabria cohort | Sequenom |  |  |  | 15 |  | 13 |  |
|  |  | Controls |  |  |  |  |  |  |  |  |  |
| Danish longevity study II | Replication | Cases | Danish longevity study II | Illumina OmniExpress | > 95% | > 1% | > 10^-4^ | 307990 | IMPUTE | 15 |  |
|  |  | Controls | GOYA III | Illumina Human610 |  |  |  |  |  |  |  |
| deCODE | Replication | Cases | deCODE  deCODE | Illumina Infinium HumanHap300/Illumina Infinium II HumanHap550-Duo/Illumina Human610/Illumina OmniExpress | > 98% | > 1% | > 10^-4^ | NA | deCODE software | 15 |  |
|  |  | Controls |  |  |  |  |  |  |  |  |  |
| German longevity study | Replication | Cases | German longevity study  German longevity study | Sequenom |  |  |  | 15 |  | 13 |  |
|  |  | Controls |  |  |  |  |  |  |  |  |  |
| Leiden 85-plus study II | Replication | Cases | Leiden 85-plus study II | Sequenom |  |  |  | 15 |  | 13 |  |
|  |  | Controls | NTR II |  |  |  |  |  |  |  |  |

MAF, minor allele frequency; HWE *P*, Hardy-Weinberg equilibrium *P*-value.

**Table S3.** Results of the conditional analysis to test for independent signals at chromosome 19q13.32.

|  |  |  | ***P*** | |
| --- | --- | --- | --- | --- |
| **SNP** | **Chr.** | **Position** | **Unadjusted** | **Adjusted for rs4420638** |
| rs6857 | 19 | 50084094 | 5.11 x 10^-17^ | 0.114 |
| rs2075650 | 19 | 50087459 | 4.46 x 10^-14^ | 0.250 |
| rs157582 | 19 | 50088059 | 8.92 x 10^-12^ | 0.188 |
| rs157580 | 19 | 50087106 | 9.50 x 10^-6^ | 0.729 |
| rs10119 | 19 | 50098513 | 7.38 x 10^-5^ | 0.768 |
| rs405509 | 19 | 50100676 | 8.51 x 10^-5^ | 0.003 |
| rs6859 | 19 | 50073874 | 1.00 x 10^-4^ | 0.108 |

**Table S4.** Results of the sex-stratified analysis of the cases ≥ 85 years of age for the 15 loci taken forward to the replication phase.

|  |  |  |  |  | **Women** | | | | **Men** | | | | **Gender difference** |
| --- | --- | --- | --- | --- | --- | --- | --- | --- | --- | --- | --- | --- | --- |
| **Locus** | **SNP** | **Chr.** | **Position** | **EA** | ***n_cases_*** | **OR** | **95% CI** | ***P*** | ***n_cases_*** | **OR** | **95% CI** | ***P*** | ***P*** |
| 1q43 | rs1625040 | 1 | 235213002 | G | 5400 | 0.87 | 0.81 - 0.93 | 3.28 x 10^-5^ | 1865 | 0.85 | 0.77 - 0.94 | 0.001 | 0.716 |
| 2q24.3 | rs6432832 | 2 | 166079072 | C | 5400 | 0.90 | 0.85 - 0.95 | 4.62 x 10^-5^ | 1865 | 0.86 | 0.80 - 0.93 | 1.43 x 10^-4^ | 0.402 |
| 4q22.2 | rs4693331 | 4 | 94760609 | C | 5400 | 0.91 | 0.87 - 0.96 | 2.16 x 10^-4^ | 1865 | 0.89 | 0.83 - 0.96 | 0.002 | 0.576 |
| 4q27 | rs13114426 | 4 | 120942533 | T | 5400 | 0.90 | 0.86 - 0.95 | 6.90 x 10^-5^ | 1865 | 0.93 | 0.86 - 1.00 | 0.056 | 0.509 |
| 5q33.3 | rs2149954 | 5 | 157753180 | T | 5400 | 1.12 | 1.07 - 1.18 | 1.35 x 10^-5^ | 1865 | 1.14 | 1.05 - 1.23 | 0.001 | 0.789 |
| 7p14.2 | rs11977641 | 7 | 36761949 | G | 5400 | 1.16 | 1.04 - 1.28 | 0.005 | 1865 | 1.09 | 0.93 - 1.27 | 0.291 | 0.515 |
| 8q13.3 | rs10957550 | 8 | 72457142 | A | 5398 | 1.14 | 1.08 - 1.21 | 4.36 x 10^-6^ | 1865 | 1.11 | 1.02 - 1.20 | 0.015 | 0.582 |
| 10q23.33 | rs4466755 | 10 | 96622243 | T | 5400 | 1.12 | 1.06 - 1.17 | 1.38 x 10^-5^ | 1865 | 1.08 | 1.00 - 1.16 | 0.044 | 0.436 |
| 12q15 | rs11834614 | 12 | 67197344 | T | 5400 | 1.15 | 1.07 - 1.23 | 9.62 x 10^-5^ | 1865 | 1.14 | 1.03 - 1.27 | 0.010 | 0.967 |
| 14q23.2 | rs2784505 | 14 | 61501766 | G | 5268 | 1.12 | 1.02 - 1.24 | 0.015 | 1865 | 1.21 | 1.06 - 1.39 | 0.005 | 0.364 |
| 17p13.1 | rs940850 | 17 | 8870805 | T | 5399 | 0.82 | 0.74 - 0.90 | 4.99 x 10^-5^ | 1865 | 0.90 | 0.78 - 1.03 | 0.135 | 0.282 |
| 17q23.2 | rs2109265 | 17 | 58307001 | G | 5400 | 0.91 | 0.87 - 0.96 | 2.50 x 10^-4^ | 1865 | 0.91 | 0.85 - 0.98 | 0.009 | 0.916 |
| 17q23.3 | rs17760362 | 17 | 58772399 | T | 5400 | 0.88 | 0.83 - 0.93 | 4.69 x 10^-6^ | 1865 | 0.88 | 0.81 - 0.96 | 0.003 | 0.910 |
| 19q13.32 | rs4420638 | 19 | 50114786 | G | 5399 | 0.72 | 0.66 - 0.78 | 2.66 x 10^-15^ | 1865 | 0.73 | 0.65 - 0.82 | 1.14 x 10^-7^ | 0.873 |
| 20q13.2 | rs8126377 | 20 | 51590254 | T | 5268 | 1.22 | 1.09 - 1.37 | 6.90 x 10^-4^ | 1723 | 1.40 | 1.17 - 1.67 | 2.25 x 10^-4^ | 0.214 |

EA, effect allele; OR, odds ratio; 95% CI, 95% confidence interval.

**Table S5.** Details of the cohorts used for the prospective analysis of rs4420638 and rs2149954.

|  |  | ***n_deaths_*** | | | | |  |  |  |
| --- | --- | --- | --- | --- | --- | --- | --- | --- | --- |
| **Study** | ***n*** | **All-cause** | **CVD** | **CAD** | **Stroke** | **Non-CVD** | **Mean age at inclusion (SD)** | **Age range** | **Mean follow-up time (SD)*** |
| Calabria cohort | 231 | 170 | NA | NA | NA | NA | 94.5 (4.8) | 85 - 105 | 5.1 (1.5) |
| Danish longevity study I + II | 960 | 542 | NA | NA | NA | NA | 96.9 (3.8) | 90 - 100 | 2.2 (2.5) |
| EGCUT | 7012 | 1247 | 605 | 271 | 94 | 621 | 51.4 (20.4) | 18 - 103 | 4.52 (1.33) |
| Leiden 85-plus study Cohort I | 628 | 627 | 256 | 65 | 84 | 371 | 89.8 (3.0) | 84 - 100 | 17.4 (NA) |
| Leiden 85-plus study Cohort II | 538 | 519 | 195 | 51 | 55 | 315 | 85.0 (NA) | NA | 13.6 (0.6) |
| LLS nonagenarians | 931 | 808 | NA | NA | NA | NA | 93.4 (2.7) | 89 - 103 | 7.5 (0.8) |
| LLS offspring + partners | 2339 | 109 | NA | NA | NA | NA | 59.1 (6.8) | 30 - 80 | 7.6 (0.9) |
| Newcastle 85+ Study | 642 | 303 | NA | NA | NA | NA | 85.0 (NA) | NA | 4.8 (0.4) |
| PROSPER | 5244 | 548 | 266 | 197 | 31 | 282 | 75.3 (3.4) | 69 - 83 | 3.2 (0.6) |
| Rotterdam Study I | 5974 | 3192 | 1031 | 162 | 292 | 2161 | 69.4 (9.1) | 55 - 99 | 12.5 (5.2) |
| TwinGene | 9604 | 517 | 284 | 131 | 68 | 233 | 65.0 (8.2) | 47 - 94 | 4.3 (1.0) |
| **Combined** | 34103 | 8582 | 2637 | 877 | 624 | 3983 |  |  |  |

CVD, cardiovascular disease; CAD, coronary artery disease. *The mean follow-up time is based on the individuals that were still alive at the end of follow-up.

**Table S6.** Results of the prospective analysis of rs4420638 and rs2149954 (all-cause mortality).

| **SNP** | **Study** | ***n*** | ***n_death_*** | **HR** | **95% CI** | ***P*** | ***I^2^ (%)*** | | ***P*_het_** | |
| --- | --- | --- | --- | --- | --- | --- | --- | --- | --- | --- |
| rs4420638 | Danish longevity study I + II | 960 | 542 | 1.09 | 0.85 - 1.40 | 1.40 | 0.489 |  | |  |
|  | EGCUT | 7012 | 1247 | 0.99 | 0.89 - 1.11 | 1.11 | 0.885 |  | |  |
|  | LLS nonagenarians | 931 | 808 | 1.24 | 1.04 - 1.47 | 1.47 | 0.017 |  | |  |
|  | LLS offspring + partners | 2339 | 109 | 1.03 | 0.63 - 1.70 | 1.70 | 0.901 |  | |  |
|  | Newcastle 85+ Study | 642 | 303 | 1.20 | 0.98 - 1.47 | 1.47 | 0.114 |  | |  |
|  | PROSPER | 5244 | 548 | 1.00 | 0.81 - 1.23 | 1.23 | 0.969 |  | |  |
|  | Rotterdam Study I | 5974 | 3192 | 1.08 | 0.99 - 1.18 | 1.18 | 0.074 |  | |  |
|  | TwinGene | 9604 | 517 | 1.07 | 0.92 - 1.26 | 1.26 | 0.376 |  | |  |
|  | **Combined** | 32706 | 7266 | 1.07 | 1.01 - 1.13 | 1.13 | 0.019 | 0.0 | | 0.658 |
| rs2149954 | Calabria cohort | 231 | 170 | 1.01 | 0.82 - 1.24 | 1.24 | 0.932 |  | |  |
|  | Danish longevity study I + II | 960 | 542 | 0.91 | 0.79 - 1.04 | 1.04 | 0.153 |  | |  |
|  | EGCUT | 7012 | 1247 | 1.03 | 0.95 - 1.12 | 1.12 | 0.492 |  | |  |
|  | Leiden 85-plus study Cohort I | 628 | 627 | 0.98 | 0.88 - 1.10 | 1.10 | 0.755 |  | |  |
|  | Leiden 85-plus study Cohort II | 538 | 519 | 0.88 | 0.77 - 0.99 | 0.99 | 0.039 |  | |  |
|  | LLS nonagenarians | 931 | 808 | 0.96 | 0.86 - 1.07 | 1.07 | 0.478 |  | |  |
|  | LLS offspring + partners | 2339 | 109 | 1.06 | 0.78 - 1.44 | 1.44 | 0.717 |  | |  |
|  | Newcastle 85+ Study | 642 | 303 | 0.84 | 0.70 - 1.01 | 1.01 | 0.047 |  | |  |
|  | PROSPER | 5244 | 548 | 0.87 | 0.77 - 0.99 | 0.99 | 0.031 |  | |  |
|  | Rotterdam Study I | 5974 | 3192 | 0.98 | 0.93 - 1.04 | 1.04 | 0.521 |  | |  |
|  | TwinGene | 9604 | 517 | 0.86 | 0.75 - 0.98 | 0.98 | 0.019 |  | |  |
|  | **Combined** | 34103 | 8582 | 0.95 | 0.93 - 0.98 | 0.98 | 0.003 | 35.8 | | 0.112 |

HR, hazard ratio; SE, standard error; 95% CI, 95% confidence interval; *I^2^*, heterogeneity statistic; *P*_het_, *P*-value for heterogeneity.

**Table S7.** Results of the prospective analysis of rs2149954 (cause-specific mortality).

| **Mortality** | **Study** | ***n*** | ***n_death_*** | **HR** | **95% CI** | ***P*** | ***I^2^ (%)*** | ***P*_het_** |
| --- | --- | --- | --- | --- | --- | --- | --- | --- |
| CVD | Leiden 85-plus Cohort II | 529 | 195 | 0.89 | 0.72 - 1.10 | 0.272 |  |  |
|  | PROSPER | 5244 | 266 | 0.78 | 0.65 - 0.94 | 0.007 |  |  |
|  | TwinGene | 9604 | 284 | 0.90 | 0.76 - 1.07 | 0.237 |  |  |
|  | **Combined** | 15377 | 745 | 0.86 | 0.77 - 0.95 | 0.004 | 0.0 | 0.485 |
| CAD | Leiden 85-plus Cohort II | 529 | 51 | 0.98 | 0.66 - 1.46 | 0.914 |  |  |
|  | PROSPER | 5244 | 197 | 0.80 | 0.65 - 0.99 | 0.036 |  |  |
|  | TwinGene | 9604 | 131 | 1.02 | 0.79 - 1.32 | 0.866 |  |  |
|  | **Combined** | 15377 | 379 | 0.90 | 0.77 - 1.04 | 0.165 | 13.9 | 0.313 |
| Stroke | Leiden 85-plus Cohort II | 529 | 55 | 0.59 | 0.39 - 0.89 | 0.013 |  |  |
|  | PROSPER | 5244 | 31 | 0.56 | 0.31 - 0.99 | 0.046 |  |  |
|  | TwinGene | 9604 | 68 | 0.65 | 0.44 - 0.94 | 0.023 |  |  |
|  | **Combined** | 15377 | 154 | 0.60 | 0.49 - 0.73 | 2.27 x 10^-7^ | 0.0 | 0.889 |
| Non-CVD | Leiden 85-plus Cohort II | 529 | 315 | 0.83 | 0.70 - 0.97 | 0.022 |  |  |
|  | PROSPER | 5244 | 282 | 0.97 | 0.81 - 1.15 | 0.685 |  |  |
|  | TwinGene | 9604 | 233 | 0.80 | 0.66 - 0.98 | 0.028 |  |  |
|  | **Combined** | 15377 | 830 | 0.86 | 0.78 - 0.95 | 0.002 | 27.4 | 0.252 |

CVD, cardiovascular disease; CAD, coronary artery disease; HR, hazard ratio; SE, standard error; 95% CI, 95% confidence interval; *I^2^*, heterogeneity statistic; *P*_het_, *P*-value for heterogeneity.

**Table S8.** Association of rs2149954 and rs4420638 with CAD and type 2 diabetes.

| **SNP** | **Phenotype** | **Study** | ***n_c_*_ases_** | ***n_controls_*** | **EA** | **OA** | **OR** | **95% CI** | ***P*** |
| --- | --- | --- | --- | --- | --- | --- | --- | --- | --- |
| rs4420638 | CAD | Schunkert *et al*. 2011 (31) | 10669 | 25397 | G | A | 1.12 | 1.05 - 1.19 | 2.14 x 10^-4^ |
| rs2149954 | CAD | Schunkert *et al*. 2011 (31) | 21931 | 62250 | T | C | 0.96 | 0.94 - 0.99 | 0.011 |
| rs4420638 | Type 2 diabetes | Morris *et al*. 2012 (32) | 10706 | 33668 | G | A | 0.88 | 0.83 - 0.92 | 3.20 x 10^-7^ |
| rs2149954 | Type 2 diabetes | Morris *et al*. 2012 (32) | 12171 | 56862 | T | C | 0.98 | 0.94 - 1.01 | 0.210 |

CAD, coronary artery disease; EA, effect allele; OA, other allele; OR: odds ratio for the effect allele; 95% CI, 95% confidence interval.

**Table S9.** Association of rs4420638 and rs2149954 with blood pressure and metabolic traits.

| **SNP** | **Phenotype** | **Study** | ***n*** | **EA** | **OA** | **β** | **95% CI** | ***P*** |
| --- | --- | --- | --- | --- | --- | --- | --- | --- |
| rs4420638 | DBP | Ehret *et al*. 2011 (33) |  |  |  |  |  | 0.032 |
| rs2149954 | DBP | Ehret *et al*. 2011 (33) |  |  |  |  |  | 3.46 x 10^-5^ |
| rs4420638 | SBP | Ehret *et al*. 2011 (33) |  |  |  |  |  | 0.297 |
| rs2149954 | SBP | Ehret *et al*. 2011 (33) |  |  |  |  |  | 6.55 x 10^-6^ |
| rs4420638 | 2hr glucose (OGTT) (adjusted for BMI) | Saxena *et al*. 2010 (34) |  | G | A | -0.009 | -0.066 - 0.048 | 0.764 |
| rs2149954 | 2hr glucose (OGTT) (adjusted for BMI) | Saxena *et al*. 2010 (34) |  | T | C | -0.019 | -0.056 - 0.018 | 0.318 |
| rs4420638 | Fasting glucose | Manning *et al*. 2012 (35) |  | G | A | -0.011 | -0.020 - -0.002 | 0.023 |
| rs2149954 | Fasting glucose | Manning *et al*. 2012 (35) |  | T | C | 0.004 | -0.002 - 0.010 | 0.226 |
| rs4420638 | Fasting insulin* | Manning *et al*. 2012 (35) |  | G | A | -0.012 | -0.021 - -0.003 | 0.014 |
| rs2149954 | Fasting insulin* | Manning *et al*. 2012 (35) |  | T | C | 0.003 | -0.004 - 0.009 | 0.398 |
| rs4420638 | Fasting proinsulin* | Strawbridge *et al.* 2011 (36) |  | G | A | -0.015 | -0.037 - 0.007 | 0.167 |
| rs2149954 | Fasting proinsulin* | Strawbridge *et al.* 2011 (36) |  | T | C | 0.001 | -0.013 - 0.015 | 0.914 |
| rs4420638 | Insulin Resistance (HOMA-IR) | Dupuis *et al*. 2010 (37) |  | G | A | -0.007 | -0.018 - 0.005 | 0.277 |
| rs2149954 | Insulin Resistance (HOMA-IR) | Dupuis *et al*. 2010 (37) |  | T | C | 0.001 | -0.007 - 0.009 | 0.751 |
| rs4420638 | β-cell activity (HOMA-B) | Dupuis *et al*. 2010 (37) |  | G | A | -0.001 | -0.011 - 0.009 | 0.853 |
| rs2149954 | β-cell activity (HOMA-B) | Dupuis *et al*. 2010 (37) |  | T | C | -0.001 | -0.008 - 0.006 | 0.754 |
| rs4420638 | Hb_1_Ac | Soranzo *et al.* 2010 (38) |  | G | A | -0.003 | -0.013 - 0.007 | 0.576 |
| rs2149954 | Hb_1_Ac | Soranzo *et al.* 2010 (38) |  | T | C | 0.000 | -0.007 - 0.007 | 0.947 |
| rs4420638 | HDL cholesterol | Teslovich *et al*. 2010 (39) | 87520 | G | A | - |  | 4.40 x 10^-21^ |
| rs2149954 | HDL cholesterol | Teslovich *et al*. 2010 (39) | 99888 | T | C | + |  | 0.745 |
| rs4420638 | LDL cholesterol | Teslovich *et al*. 2010 (39) | 83209 | G | A | + |  | 8.72 x 10^-147^ |
| rs2149954 | LDL cholesterol | Teslovich *et al*. 2010 (39) | 95442 | T | C | - |  | 0.436 |
| rs4420638 | Total cholesterol | Teslovich *et al*. 2010 (39) | 87766 | G | A | + |  | 5.20 x 10^-111^ |
| rs2149954 | Total cholesterol | Teslovich *et al*. 2010 (39) | 100172 | T | C | - |  | 0.432 |
| rs4420638 | Triglycerides | Teslovich *et al*. 2010 (39) | 84180 | G | A | + |  | 5.44 x 10^-22^ |
| rs2149954 | Triglycerides | Teslovich *et al*. 2010 (39) | 96586 | T | C | + |  | 0.913 |

DBP, diastolic blood pressure; SBP, systolic blood pressure; OGTT, oral glucose tolerance test; BMI, body mass index; HOMA-IR, homeostasis model of assessment-insulin resistance; HOMA-B, homeostasis model of assessment-β-cell activity; Hb_1_Ac, hemoglobin A1c; HDL, high-density lipoprotein; LDL, low-density lipoprotein; EA, effect allele; OA, other allele; SE, standard error; 95% CI, 95% confidence interval. *Natural log transformed parameter was used for the analysis.

**Table S10.** Results of the prospective analysis of rs2149954 adjusted for blood pressure.

| **Mortality** | **Study** | **Analysis** | ***n*** | ***n*_death_** | **HR** | **95% CI** | ***P*** |
| --- | --- | --- | --- | --- | --- | --- | --- |
| All-cause | Leiden 85-plus Cohort II | Unadjusted | 538 | 519 | 0.88 | 0.77 - 0.99 | 0.039 |
|  | Leiden 85-plus Cohort II | Adjusted for SBP | 536 | 517 | 0.85 | 0.75 - 0.96 | 0.011 |
|  | Leiden 85-plus Cohort II | Adjusted for DBP | 536 | 517 | 0.86 | 0.76 - 0.97 | 0.018 |
|  | PROSPER | Unadjusted | 5244 | 548 | 0.87 | 0.77 - 0.99 | 0.031 |
|  | PROSPER | Adjusted for SBP | 5244 | 548 | 0.87 | 0.77 - 0.99 | 0.029 |
|  | PROSPER | Adjusted for DBP | 5244 | 548 | 0.87 | 0.77 - 0.99 | 0.030 |
| CVD | Leiden 85-plus Cohort II | Unadjusted | 529 | 195 | 0.89 | 0.72 - 1.10 | 0.272 |
|  | Leiden 85-plus Cohort II | Adjusted for SBP | 527 | 194 | 0.89 | 0.72 - 1.10 | 0.275 |
|  | Leiden 85-plus Cohort II | Adjusted for DBP | 527 | 194 | 0.89 | 0.72 - 1.09 | 0.253 |
|  | PROSPER | Unadjusted | 5244 | 266 | 0.78 | 0.65 - 0.94 | 0.007 |
|  | PROSPER | Adjusted for SBP | 5244 | 266 | 0.78 | 0.65 - 0.93 | 0.007 |
|  | PROSPER | Adjusted for DBP | 5244 | 266 | 0.78 | 0.65 - 0.94 | 0.007 |
| CAD | Leiden 85-plus Cohort II | Unadjusted | 529 | 51 | 0.98 | 0.66 - 1.46 | 0.914 |
|  | Leiden 85-plus Cohort II | Adjusted for SBP | 527 | 51 | 0.94 | 0.63 - 1.41 | 0.780 |
|  | Leiden 85-plus Cohort II | Adjusted for DBP | 527 | 51 | 0.96 | 0.65 - 1.44 | 0.859 |
|  | PROSPER | Unadjusted | 5244 | 197 | 0.80 | 0.65 - 0.99 | 0.036 |
|  | PROSPER | Adjusted for SBP | 5244 | 197 | 0.79 | 0.64 - 0.99 | 0.033 |
|  | PROSPER | Adjusted for DBP | 5244 | 197 | 0.80 | 0.64 - 0.99 | 0.034 |
| Stroke | Leiden 85-plus Cohort II | Unadjusted | 529 | 55 | 0.59 | 0.39 - 0.89 | 0.013 |
|  | Leiden 85-plus Cohort II | Adjusted for SBP | 527 | 54 | 0.59 | 0.39 - 0.90 | 0.015 |
|  | Leiden 85-plus Cohort II | Adjusted for DBP | 527 | 54 | 0.59 | 0.39 - 0.91 | 0.015 |
|  | PROSPER | Unadjusted | 5244 | 31 | 0.56 | 0.31 - 0.99 | 0.046 |
|  | PROSPER | Adjusted for SBP | 5244 | 31 | 0.56 | 0.31 - 0.98 | 0.046 |
|  | PROSPER | Adjusted for DBP | 5244 | 31 | 0.56 | 0.31 - 0.99 | 0.046 |
| Non-CVD | Leiden 85-plus Cohort II | Unadjusted | 529 | 315 | 0.83 | 0.70 - 0.97 | 0.022 |
|  | Leiden 85-plus Cohort II | Adjusted for SBP | 527 | 314 | 0.79 | 0.67 - 0.93 | 0.004 |
|  | Leiden 85-plus Cohort II | Adjusted for DBP | 527 | 314 | 0.80 | 0.68 - 0.95 | 0.009 |
|  | PROSPER | Unadjusted | 5244 | 282 | 0.97 | 0.81 - 1.15 | 0.685 |
|  | PROSPER | Adjusted for SBP | 5244 | 282 | 0.96 | 0.81 - 1.15 | 0.679 |
|  | PROSPER | Adjusted for DBP | 5244 | 282 | 0.97 | 0.81 - 1.15 | 0.685 |

CVD, cardiovascular disease; CAD, coronary artery disease; HR, hazard ratio; SE, standard error; 95% CI, 95% confidence interval.

**Table S11.** Results of the GRAIL analysis of SNPs that showed moderate evidence for association (*P ≤* 1 x 10^-4^) with survival ≥ 90 years of age in the discovery phase analysis.

| **SNP** | ***P* discovery phase ≥ 90 years** | **Most significant gene** | ***P*** | ***P* (FDR)** | **Other genes within the locus (*P*)** |
| --- | --- | --- | --- | --- | --- |
| rs9452252 | 2.42 x 10^-5^ | *EPHA7* | 0.004 | 0.004 |  |
| rs712297 | 7.56 x 10^-5^ | *NPAS3* | 0.004 | 0.008 | *EGLN3* (0.317) |
| rs2060478 | 6.35 x 10^-6^ | *GRID2* | 0.009 | 0.009 |  |
| rs10501586 | 6.13 x 10^-5^ | *DLG2* | 0.011 | 0.011 |  |
| rs1525758 | 2.16 x 10^-6^ | *NTN1* | 0.014 | 0.014 |  |
| rs7808864 | 5.47 x 10^-5^ | *SEMA3A* | 0.023 | 0.023 |  |
| rs10901498 | 2.14 x 10^-5^ | *ADAM12* | 0.021 | 0.042 | *FANK1* (0.413) |
| rs525798 | 1.65 x 10^-5^ | *GRIK3* | 0.058 | 0.058 |  |
| rs11977641 | 7.31 x 10^-6^ | *ELMO1* | 0.030 | 0.059 | *AOAH* (0.911) |
| rs2836477 | 4.93 x 10^-5^ | *ERG* | 0.060 | 0.060 |  |
| rs6929631 | 7.33 x 10^-5^ | *UTRN* | 0.062 | 0.062 |  |
| rs13386398 | 2.37 x 10^-5^ | *OSR1* | 0.070 | 0.070 |  |
| rs10404 | 9.25 x 10^-5^ | *ADAM19* | 0.031 | 0.090 | *CYFIP2* (0.249), *ICHTHYIN* (0.591) |
| rs960434 | 9.27 x 10^-5^ | *NEUROD6* | 0.094 | 0.094 |  |
| rs2109265 | 3.34 x 10^-6^ | *TANC2* | 0.024 | 0.094 | *TLK2* (0.787), *MARCH10* (0.848), *MRC2* (0.859) |
| rs7641320 | 8.31 x 10^-5^ | *MITF* | 0.122 | 0.122 |  |
| rs4420638 | 4.09 x 10^-21^ | *APOE* | 0.044 | 0.126 | *PVRL2* (0.049), *APOC1* (0.209) |
| rs821551 | 1.53 x 10^-5^ | *EFNA1* | 0.005 | 0.146 | *RIT1* (0.064), *KIAA0907* (0.074), *GON4L* (0.100), *HCN3* (0.139), *CLK2* (0.148), *SYT11* (0.216), *GBA* (0.223), *ARHGEF2* (0.229), *ASH1L* (0.319), *GBAP* (0.349), *C1orf2* (0.479), *MSTO1* (0.484), *MUC1* (0.591), *PKLR* (0.604), *RAG1AP1* (0.671), *TRIM46* (0.704), *YY1AP1* (0.709), *KRTCAP2* (0.730), *RXFP4* (0.757), *THBS3* (0.777), *DPM3* (0.782), *SSR2* (0.793), *C1orf104* (0.852), *SCAMP3* (0.863), *RUSC1* (0.870), *UBQLN4* (0.886), *MTX1* (0.909), *DAP3* (0.920), *SNORA42* (0.921), *FDPS* (0.942) |
| rs10508899 | 8.26 x 10^-5^ | *ARHGAP22* | 0.031 | 0.146 | *PTPN20B* (0.061), *PTPN20A* (0.096), *FRMPD2* (0.265), *MAPK8* (0.356) |
| rs7701003 | 1.83 x 10^-6^ | *EBF1* | 0.157 | 0.157 |  |
| rs1911334 | 5.41 x 10^-5^ | *CTNNA3* | 0.166 | 0.166 |  |
| rs13407773 | 3.67 x 10^-5^ | *DNER* | 0.047 | 0.175 | *FBXO36* (0.564), *TRIP12* (0.678), *SLC16A14* (0.707) |
| rs7970906 | 5.28 x 10^-5^ | *CHFR* | 0.068 | 0.189 | *GOLGA3* (0.856), *KIAA0692* (1.000) |
| rs7617481 | 3.53 x 10^-5^ | *CPNE4* | 0.191 | 0.191 |  |
| rs306229 | 8.32 x 10^-5^ | *PHLPP* | 0.202 | 0.202 |  |
| rs2208520 | 2.68 x 10^-5^ | *PTPRT* | 0.114 | 0.214 | *CHD6* (0.680) |
| rs868022 | 4.18 x 10^-5^ | *GJB6* | 0.041 | 0.224 | *GJB2* (0.045), *IFT88* (0.222), *GJA3* (0.313), *ZMYM2* (0.505), *CRYL1* (0.520) |
| rs1625040 | 6.53 x 10^-6^ | *ACTN2* | 0.085 | 0.235 | *MTR* (0.116), *RYR2* (0.247) |
| rs11622644 | 5.17 x 10^-6^ | *SYT16* | 0.238 | 0.238 |  |
| rs36954 | 7.48 x 10^-5^ | *KCNN2* | 0.266 | 0.266 |  |
| rs11188105 | 7.65 x 10^-6^ | *CYP2C19* | 0.054 | 0.285 | *CYP2C9* (0.057), *TBC1D12* (0.260), *HELLS* (0.262), *CYP2C8* (0.362), *CYP2C18* (0.805) |
| rs156828 | 3.21 x 10^-5^ | *ARL15* | 0.306 | 0.306 |  |
| rs2177795 | 3.11 x 10^-5^ | *TNS3* | 0.320 | 0.320 |  |
| rs4706776 | 2.94 x 10^-5^ | *LCA5* | 0.122 | 0.322 | *PHIP* (0.400), *HMGN3* (0.460) |
| rs7711971 | 2.70 x 10^-5^ | *ADRA1B* | 0.123 | 0.325 | *PWWP2A* (0.311), *TTC1* (0.533) |
| rs2741511 | 9.63 x 10^-5^ | *RBPJL* | 0.050 | 0.368 | *WFDC5* (0.641), *MATN4* (0.649), *WFDC12* (0.672), *SLPI* (0.745), *KCNS1* (0.781), *SEMG2* (0.829), *PI3* (0.904), *SEMG1* (0.949) |
| rs9833196 | 8.15 x 10^-5^ | *LXN* | 0.077 | 0.381 | *SCHIP1* (0.256), *RARRES1* (0.649), *MFSD1* (0.834), *IQCJ* (0.931), *GFM1* (0.992) |
| rs6682217 | 9.47 x 10^-5^ | *IGSF21* | 0.214 | 0.382 | *ACTL8* (0.587) |
| rs2389922 | 3.89 x 10^-7^ | *PDE5A* | 0.221 | 0.394 | *MAD2L1* (0.350) |
| rs1565978 | 4.71 x 10^-5^ | *FHOD3* | 0.395 | 0.395 |  |
| rs17076080 | 3.09 x 10^-5^ | *NKX2-5* | 0.158 | 0.404 | *STC2* (0.208), *FAM44B* (0.291) |
| rs6603919 | 1.97 x 10^-5^ | *TAS1R2* | 0.438 | 0.438 |  |
| rs4514016 | 8.91 x 10^-5^ | *SAMD12* | 0.444 | 0.444 |  |
| rs930792 | 4.00 x 10^-5^ | *FAM130A2* | 0.254 | 0.444 | *TTC21B* (0.417) |
| rs3802030 | 9.66 x 10^-5^ | *PFTK1* | 0.499 | 0.499 |  |
| rs915049 | 8.31 x 10^-6^ | *TSHZ2* | 0.563 | 0.563 |  |
| rs4629011 | 4.16 x 10^-5^ | *C17orf92* | 0.099 | 0.567 | *HOXB13* (0.151), *GIP* (0.722), *TTLL6* (0.781), *UBE2Z* (0.891), *ATP5G1* (0.909), *CALCOCO2* (0.922), *SNF8* (0.976) |
| rs2835378 | 6.14 x 10^-5^ | *CLDN14* | 0.580 | 0.580 |  |
| rs13125491 | 5.04 x 10^-5^ | *CCRN4L* | 0.378 | 0.613 | *ELF2* (0.517) |
| rs2145288 | 4.72 x 10^-5^ | *GNAS* | 0.379 | 0.614 | *NESPAS* (0.599) |
| rs7440402 | 7.31 x 10^-5^ | *CCDC149* | 0.648 | 0.648 |  |
| rs4526676 | 4.04 x 10^-5^ | *LYZL1* | 0.660 | 0.660 |  |
| rs10457481 | 6.81 x 10^-5^ | *C6orf173* | 0.661 | 0.661 |  |
| rs951351 | 8.90 x 10^-5^ | *ITGB3* | 0.248 | 0.680 | *C17orf57* (0.440), *KPNB1* (0.589), *NPEPPS* (0.816) |
| rs11785849 | 3.09 x 10^-5^ | *ST3GAL1* | 0.759 | 0.759 |  |
| rs12794996 | 4.93 x 10^-5^ | *PMP22CD* | 0.319 | 0.784 | *ZNF202* (0.542), *OR6M1* (0.976), *OR6X1* (0.979) |
| rs11834614 | 9.94 x 10^-6^ | *NUP107* | 0.405 | 0.789 | *RAP1B* (0.495), *SLC35E3* (0.711) |
| rs2280654 | 7.72 x 10^-5^ | *TSPAN13* | 0.499 | 0.874 | AGR2 (0.521), AGR3 (0.941) |
| rs9327463 | 9.69 x 10^-5^ | *FLJ33630* | 0.978 | 0.978 |  |
| rs12613610 | 6.86 x 10^-5^ | *CD8B* | 0.654 | 0.998 | *RMND5A* (0.656), *PLGLB2* (0.676), *CD8A* (0.706), *LOC285074* (0.775), *RGPD1* (0.799) |
| rs10967226 | 8.73 x 10^-5^ |  |  | NA |  |
| rs11043346 | 8.69 x 10^-5^ |  |  | NA |  |
| rs16852818 | 4.44 x 10^-5^ |  |  | NA |  |
| rs6958623 | 6.91 x 10^-5^ |  |  | NA |  |
| rs8090147 | 8.32 x 10^-5^ |  |  | NA |  |

NA, not applicable.

**Table S12.** Functional implications of SNPs on chromosome 5q33.3.

| **SNP** | **Position (b37)** | **r^2^** | **D'** | **Ref** | **Alt** | **MAF CEU** | **Enhancer histone marks** | **DNase** | **Proteins bound** | **Motifs changed** | **Regulome DB Score** |
| --- | --- | --- | --- | --- | --- | --- | --- | --- | --- | --- | --- |
| rs7715501 | 157802270 | 0.87 | 0.94 | T | A | 0.340 |  |  |  | TCF4 | No Data |
| rs7442660 | 157803630 | 0.99 | 0.99 | G | A | 0.330 |  |  |  | 5 altered motifs | 6 |
| rs9313772 | 157804457 | 0.94 | 1.00 | C | T | 0.320 |  |  |  | Mef2,Pbx3 | No Data |
| rs7712354 | 157806250 | 0.94 | 1.00 | G | A | 0.320 |  |  |  | Znf143 | 5 |
| rs1952652 | 157811088 | 0.95 | 1.00 | A | T | 0.320 | 5 cell types |  |  | 21 altered motifs | 6 |
| rs12187074 | 157811935 | 1.00 | 1.00 | C | G | 0.330 | 5 cell types | HRGEC |  | Zfp410 | 5 |
| rs12188031 | 157812020 | 0.99 | 1.00 | T | C | 0.340 | 5 cell types | 11 cell types |  | 4 altered motifs | 5 |
| rs6867081 | 157813013 | 0.95 | 1.00 | T | C | 0.320 | 5 cell types |  |  | CDP,Foxp1,Hdx | No Data |
| rs6863179 | 157813148 | 0.95 | 1.00 | A | G | 0.320 | 4 cell types | LNCaP | FOXA1 | 7 altered motifs | 3a |
| rs12716337 | 157814228 | 0.95 | 0.99 | T | C | 0.340 |  |  |  | PLAG1,STAT | No Data |
| rs7719885 | 157816127 | 0.99 | 1.00 | A | G | 0.340 |  |  | GATA2 | Nkx2 | 5 |
| rs7737275 | 157816142 | 1.00 | 1.00 | G | A | 0.330 |  |  | GATA2 | 7 altered motifs | 5 |
| rs7720317 | 157816343 | 0.98 | 0.99 | A | G | 0.330 |  |  |  | 4 altered motifs | No Data |
| rs10076730 | 157816992 | 0.95 | 1.00 | T | C | 0.340 | NHLF, Huvec | 6 cell types |  |  | 5 |
| rs13178245 | 157817293 | 0.95 | 1.00 | A | G | 0.350 | NHLF, Huvec, HMEC |  |  | RXRA,p300 | No Data |
| rs11960210 | 157817634 | 0.95 | 1.00 | T | C | 0.340 | 5 cell types | 7 cell types | 6 bound proteins | LBP-9,RORalpha1 | 4 |
| rs10045018 | 157818018 | 0.95 | 1.00 | A | G | 0.340 | 5 cell types |  |  | Pax-6,Pou2f2,Pou3f3 | 6 |
| rs10042238 | 157818150 | 1.00 | 1.00 | C | G | 0.330 | Huvec |  |  | Foxp1,PTF1-beta | 6 |
| rs10051330 | 157819624 | 0.95 | 1.00 | A | G | 0.320 | Huvec |  |  | 4 altered motifs | No Data |
| rs7721599 | 157819991 | 1.00 | 1.00 | C | T | 0.330 | Huvec, NHLF, GM12878 |  |  | Mef2,RXRA | No Data |
| **rs2149954** | **157820602** |  |  | C | T | 0.330 | Huvec, GM12878 | 9 cell types | 4 bound proteins | COMP1 | 4 |
| rs1958603 | 157822114 | 0.95 | 1.00 | T | A | 0.340 |  |  |  | 5 altered motifs | 6 |
| rs7700842 | 157824183 | 0.95 | 0.99 | T | C | 0.340 |  |  |  | CEBPB,HMG-IY | 6 |
| rs7701003 | 157824481 | 0.94 | 0.99 | A | G | 0.340 |  | RPTEC |  | Ets,RFX5,Zec | 5 |
| rs4704775 | 157824556 | 0.94 | 1.00 | G | A | 0.320 |  |  |  | AIRE,Foxm1 | No Data |

r^2^ and D’, linkage disequilibrium with rs2149954; Ref, reference allele; Alt, other allele; MAF CEU, minor allele frequency based on 1000 Genomes CEU Phase 1 data; Enhancer histone marks, regulatory chromatin states based on ENCODE data; DNase, DNase hypersensitivity based on ENCODE data; Proteins bound, proteins bound by chromatin immunoprecipitation based on ENCODE data; Motifs changed, altered regulatory motifs.

**Supplemental references**

1. Rea, I.M., Myint, P.K., Mueller, H., Murphy, A., Archbold, G.P., McNulty, H., Patterson, C.C. (2009) Nature or nurture; BMI and blood pressure at 90. Findings from the Belfast Elderly Longitudinal Free-living Aging STudy (BELFAST). *Age (Dordr. )*, **31**, 261-267.

2. Folstein, M.F., Folstein, S.E., McHugh, P.R. (1975) "Mini-mental state". A practical method for grading the cognitive state of patients for the clinician. *J Psychiatr. Res.*, **12**, 189-198.

3. Ligthart, G.J., Corberand, J.X., Fournier, C., Galanaud, P., Hijmans, W., Kennes, B., Muller-Hermelink, H.K., Steinmann, G.G. (1984) Admission criteria for immunogerontological studies in man: the SENIEUR protocol. *Mech. Ageing Dev.*, **28**, 47-55.

4. De Rango, F., Montesanto, A., Berardelli, M., Mazzei, B., Mari, V., Lattanzio, F., Corsonello, A., Passarino, G. (2011) To grow old in southern Italy: a comprehensive description of the old and oldest old in Calabria. *Gerontology*, **57**, 327-334.

5. Blanche, H., Cabanne, L., Sahbatou, M., Thomas, G. (2001) A study of French centenarians: are ACE and APOE associated with longevity? *C. R. Acad. Sci. III*, **324**, 129-135.

6. Christensen, K., McGue, M., Petersen, I., Jeune, B., Vaupel, J.W. (2008) Exceptional longevity does not result in excessive levels of disability. *Proc. Natl. Acad. Sci. U. S. A*, **105**, 13274-13279.

7. Andersen-Ranberg, K., Schroll, M., Jeune, B. (2001) Healthy centenarians do not exist, but autonomous centenarians do: a population-based study of morbidity among Danish centenarians. *J Am. Geriatr. Soc.*, **49**, 900-908.

8. McGue, M., Christensen, K. (2007) Social activity and healthy aging: a study of aging Danish twins. *Twin. Res. Hum. Genet.*, **10**, 255-265.

9. Miller, S.A., Dykes, D.D., Polesky, H.F. (1988) A simple salting out procedure for extracting DNA from human nucleated cells. *Nucleic Acids Res.*, **16**, 1215.

10. Robine, J.M., Cheung, S.L., Saito, Y., Jeune, B., Parker, M.G., Herrmann, F.R. (2010) Centenarians Today: New Insights on Selection from the 5-COOP Study. *Curr. Gerontol. Geriatr. Res.*, **2010**, 120354.

11. Gudmundsson, H., Gudbjartsson, D.F., Frigge, M., Gulcher, J.R., Stefansson, K. (2000) Inheritance of human longevity in Iceland. *Eur. J. Hum. Genet.*, **8**, 743-749.

12. Skytthe, A., Valensin, S., Jeune, B., Cevenini, E., Balard, F., Beekman, M., Bezrukov, V., Blanche, H., Bolund, L., Broczek, K.*, et al.* (2011) Design, recruitment, logistics, and data management of the GEHA (Genetics of Healthy Ageing) project. *Exp. Gerontol.*, **46**, 934-945.

13. Nebel, A., Croucher, P.J., Stiegeler, R., Nikolaus, S., Krawczak, M., Schreiber, S. (2005) No association between microsomal triglyceride transfer protein (MTP) haplotype and longevity in humans. *Proc. Natl. Acad. Sci. U. S. A*, **102**, 7906-7909.

14. Krawczak, M., Nikolaus, S., von, E.H., Croucher, P.J., El Mokhtari, N.E., Schreiber, S. (2006) PopGen: population-based recruitment of patients and controls for the analysis of complex genotype-phenotype relationships. *Community Genet*, **9**, 55-61.

15. Paternoster, L., Evans, D.M., Nohr, E.A., Holst, C., Gaborieau, V., Brennan, P., Gjesing, A.P., Grarup, N., Witte, D.R., Jorgensen, T.*, et al.* (2011) Genome-wide population-based association study of extremely overweight young adults--the GOYA study. *PLoS. One.*, **6**, e24303.

16. Bootsma-van der Wiel, A., van Exel, E., de Craen, A.J., Gussekloo, J., Lagaay, A.M., Knook, D.L., Westendorp, R.G. (2002) A high response is not essential to prevent selection bias: results from the Leiden 85-plus study. *J Clin. Epidemiol.*, **55**, 1119-1125.

17. Weverling-Rijnsburger, A.W., Blauw, G.J., Lagaay, A.M., Knook, D.L., Meinders, A.E., Westendorp, R.G. (1997) Total cholesterol and risk of mortality in the oldest old. *Lancet*, **350**, 1119-1123.

18. Schoenmaker, M., de Craen, A.J., de Meijer, P.H., Beekman, M., Blauw, G.J., Slagboom, P.E., Westendorp, R.G. (2006) Evidence of genetic enrichment for exceptional survival using a family approach: the Leiden Longevity Study. *Eur. J Hum Genet*, **14**, 79-84.

19. Beekman, M., Blauw, G.J., Houwing-Duistermaat, J.J., Brandt, B.W., Westendorp, R.G., Slagboom, P.E. (2006) Chromosome 4q25, microsomal transfer protein gene, and human longevity: novel data and a meta-analysis of association studies. *J Gerontol. A Biol. Sci. Med. Sci.*, **61**, 355-362.

20. Collerton, J., Davies, K., Jagger, C., Kingston, A., Bond, J., Eccles, M.P., Robinson, L.A., Martin-Ruiz, C., von, Z.T., James, O.F., Kirkwood, T.B. (2009) Health and disease in 85 year olds: baseline findings from the Newcastle 85+ cohort study. *BMJ*, **339**, b4904.

21. Boomsma, D.I., Willemsen, G., Sullivan, P.F., Heutink, P., Meijer, P., Sondervan, D., Kluft, C., Smit, G., Nolen, W.A., Zitman, F.G.*, et al.* (2008) Genome-wide association of major depression: description of samples for the GAIN Major Depressive Disorder Study: NTR and NESDA biobank projects. *Eur. J Hum. Genet.*, **16**, 335-342.

22. Willemsen, G., Vink, J.M., Abdellaoui, A., den, B.A., van Beek, J.H., Draisma, H.H., van, D.J., van 't, E.D., Geels, L.M., van, L.R.*, et al.* (2013) The Adult Netherlands Twin Register: Twenty-Five Years of Survey and Biological Data Collection. *Twin. Res. Hum. Genet.*, 1-11.

23. Shepherd, J., Blauw, G.J., Murphy, M.B., Cobbe, S.M., Bollen, E.L., Buckley, B.M., Ford, I., Jukema, J.W., Hyland, M., Gaw, A.*, et al.* (1999) The design of a prospective study of Pravastatin in the Elderly at Risk (PROSPER). PROSPER Study Group. PROspective Study of Pravastatin in the Elderly at Risk. *Am. J. Cardiol.*, **84**, 1192-1197.

24. Shepherd, J., Blauw, G.J., Murphy, M.B., Bollen, E.L., Buckley, B.M., Cobbe, S.M., Ford, I., Gaw, A., Hyland, M., Jukema, J.W.*, et al.* (2002) Pravastatin in elderly individuals at risk of vascular disease (PROSPER): a randomised controlled trial. *Lancet*, **360**, 1623-1630.

25. Trompet, S., de Craen, A.J., Postmus, I., Ford, I., Sattar, N., Caslake, M., Stott, D.J., Buckley, B.M., Sacks, F., Devlin, J.J.*, et al.* (2011) Replication of LDL GWAs hits in PROSPER/PHASE as validation for future (pharmaco)genetic analyses. *BMC. Med. Genet.*, **12**, 131.

26. Hofman, A., van Duijn, C.M., Franco, O.H., Ikram, M.A., Janssen, H.L., Klaver, C.C., Kuipers, E.J., Nijsten, T.E., Stricker, B.H., Tiemeier, H.*, et al.* (2011) The Rotterdam Study: 2012 objectives and design update. *Eur. J Epidemiol.*, **26**, 657-686.

27. Hercberg, S., Galan, P., Preziosi, P., Roussel, A.M., Arnaud, J., Richard, M.J., Malvy, D., Paul-Dauphin, A., Briancon, S., Favier, A. (1998) Background and rationale behind the SU.VI.MAX Study, a prevention trial using nutritional doses of a combination of antioxidant vitamins and minerals to reduce cardiovascular diseases and cancers. SUpplementation en VItamines et Mineraux AntioXydants Study. *Int. J Vitam. Nutr. Res.*, **68**, 3-20.

28. Rahman, I., Bennet, A.M., Pedersen, N.L., de, F.U., Svensson, P., Magnusson, P.K. (2009) Genetic dominance influences blood biomarker levels in a sample of 12,000 Swedish elderly twins. *Twin. Res. Hum Genet*, **12**, 286-294.

29. Pruim, R.J., Welch, R.P., Sanna, S., Teslovich, T.M., Chines, P.S., Gliedt, T.P., Boehnke, M., Abecasis, G.R., Willer, C.J. (2010) LocusZoom: regional visualization of genome-wide association scan results. *Bioinformatics.*, **26**, 2336-2337.

30. Sebastiani, P., Solovieff, N., Dewan, A.T., Walsh, K.M., Puca, A., Hartley, S.W., Melista, E., Andersen, S., Dworkis, D.A., Wilk, J.B.*, et al.* (2012) Genetic signatures of exceptional longevity in humans. *PLoS One*, **7**, e29848.

31. Schunkert, H., Konig, I.R., Kathiresan, S., Reilly, M.P., Assimes, T.L., Holm, H., Preuss, M., Stewart, A.F., Barbalic, M., Gieger, C.*, et al.* (2011) Large-scale association analysis identifies 13 new susceptibility loci for coronary artery disease. *Nat. Genet.*, **43**, 333-338.

32. Morris, A.P., Voight, B.F., Teslovich, T.M., Ferreira, T., Segre, A.V., Steinthorsdottir, V., Strawbridge, R.J., Khan, H., Grallert, H., Mahajan, A.*, et al.* (2012) Large-scale association analysis provides insights into the genetic architecture and pathophysiology of type 2 diabetes. *Nat. Genet.*, **44**, 981-990.

33. Ehret, G.B., Munroe, P.B., Rice, K.M., Bochud, M., Johnson, A.D., Chasman, D.I., Smith, A.V., Tobin, M.D., Verwoert, G.C., Hwang, S.J.*, et al.* (2011) Genetic variants in novel pathways influence blood pressure and cardiovascular disease risk. *Nature*, **478**, 103-109.

34. Saxena, R., Hivert, M.F., Langenberg, C., Tanaka, T., Pankow, J.S., Vollenweider, P., Lyssenko, V., Bouatia-Naji, N., Dupuis, J., Jackson, A.U.*, et al.* (2010) Genetic variation in GIPR influences the glucose and insulin responses to an oral glucose challenge. *Nat. Genet.*, **42**, 142-148.

35. Manning, A.K., Hivert, M.F., Scott, R.A., Grimsby, J.L., Bouatia-Naji, N., Chen, H., Rybin, D., Liu, C.T., Bielak, L.F., Prokopenko, I.*, et al.* (2012) A genome-wide approach accounting for body mass index identifies genetic variants influencing fasting glycemic traits and insulin resistance. *Nat. Genet.*, **44**, 659-669.

36. Strawbridge, R.J., Dupuis, J., Prokopenko, I., Barker, A., Ahlqvist, E., Rybin, D., Petrie, J.R., Travers, M.E., Bouatia-Naji, N., Dimas, A.S.*, et al.* (2011) Genome-wide association identifies nine common variants associated with fasting proinsulin levels and provides new insights into the pathophysiology of type 2 diabetes. *Diabetes*, **60**, 2624-2634.

37. Dupuis, J., Langenberg, C., Prokopenko, I., Saxena, R., Soranzo, N., Jackson, A.U., Wheeler, E., Glazer, N.L., Bouatia-Naji, N., Gloyn, A.L.*, et al.* (2010) New genetic loci implicated in fasting glucose homeostasis and their impact on type 2 diabetes risk. *Nat. Genet.*, **42**, 105-116.

38. Soranzo, N., Sanna, S., Wheeler, E., Gieger, C., Radke, D., Dupuis, J., Bouatia-Naji, N., Langenberg, C., Prokopenko, I., Stolerman, E.*, et al.* (2010) Common variants at 10 genomic loci influence hemoglobin A(1)(C) levels via glycemic and nonglycemic pathways. *Diabetes*, **59**, 3229-3239.

39. Teslovich, T.M., Musunuru, K., Smith, A.V., Edmondson, A.C., Stylianou, I.M., Koseki, M., Pirruccello, J.P., Ripatti, S., Chasman, D.I., Willer, C.J.*, et al.* (2010) Biological, clinical and population relevance of 95 loci for blood lipids. *Nature*, **466**, 707-713.
